# Cis-regulatory somatic mutations and gene-expression alteration in B-cell lymphomas

**DOI:** 10.1186/s13059-015-0648-7

**Published:** 2015-04-23

**Authors:** Anthony Mathelier, Calvin Lefebvre, Allen W Zhang, David J Arenillas, Jiarui Ding, Wyeth W Wasserman, Sohrab P Shah

**Affiliations:** Department of Medical Genetics, Centre for Molecular Medicine and Therapeutics, Child and Family Research Institute, University of British Columbia, 950 West 28th Avenue, V5Z 4H4, Vancouver, BC Canada; Department of Molecular Oncology, British Columbia Cancer Agency, Vancouver, V5Z 1L3 BC Canada; Bioinformatics Graduate Program, University of British Columbia, Vancouver, V5Z 1L3 BC Canada; Department of Computer Science, University of British Columbia, Vancouver, V6T 1Z4 BC Canada; Department of Pathology and Laboratory Medicine, University of British Columbia, Vancouver, G227-2211 BC Canada

## Abstract

**Background:**

With the rapid increase of whole-genome sequencing of human cancers, an important opportunity to analyze and characterize somatic mutations lying within *cis*-regulatory regions has emerged. A focus on protein-coding regions to identify nonsense or missense mutations disruptive to protein structure and/or function has led to important insights; however, the impact on gene expression of mutations lying within *cis*-regulatory regions remains under-explored. We analyzed somatic mutations from 84 matched tumor-normal whole genomes from B-cell lymphomas with accompanying gene expression measurements to elucidate the extent to which these cancers are disrupted by *cis*-regulatory mutations.

**Results:**

We characterize mutations overlapping a high quality set of well-annotated transcription factor binding sites (TFBSs), covering a similar portion of the genome as protein-coding exons. Our results indicate that *cis*-regulatory mutations overlapping predicted TFBSs are enriched in promoter regions of genes involved in apoptosis or growth/proliferation. By integrating gene expression data with mutation data, our computational approach culminates with identification of *cis*-regulatory mutations most likely to participate in dysregulation of the gene expression program. The impact can be measured along with protein-coding mutations to highlight key mutations disrupting gene expression and pathways in cancer.

**Conclusions:**

Our study yields specific genes with disrupted expression triggered by genomic mutations in either the coding or the regulatory space. It implies that mutated regulatory components of the genome contribute substantially to cancer pathways. Our analyses demonstrate that identifying genomically altered *cis*-regulatory elements coupled with analysis of gene expression data will augment biological interpretation of mutational landscapes of cancers.

**Electronic supplementary material:**

The online version of this article (doi:10.1186/s13059-015-0648-7) contains supplementary material, which is available to authorized users.

## Background

Tumor genome analyses for mutation and cancer gene discovery have focused primarily on the protein-coding exons, spanning approximately 2% of the genome, as they are readily interpreted and easy to delineate. Large-scale consortia such as The Cancer Genome Atlas have completed interrogation of the protein-coding genome and revealed the mutation prevalence of previously known cancer genes across the major tumor types, in addition to discovery of previously unknown biological processes disrupted by somatic mutations [1]. However, synthesis of the vast analyses of The Cancer Genome Atlas projects has revealed a discovery gap in the search for new cancer genes [2]. We assert this gap can be partially filled through analysis of the non-coding genome. In germline genetic disease studies, evidences for the impact of variations in the non-coding space of the human genome, including in *cis*-regulatory loci, on human phenotypes have accumulated over recent decades [3]. Gene-expression regulation occurs through multiple layers, one of them mediated by DNA-binding transcription factors (TFs). Disruption of sequence-specific TF binding sites (TFBSs) has been linked to numerous genetic disorders. For example, mutation within a HNF4A binding site upstream of the Factor IX gene is associated with hemophilia B Leyden [4], and alteration of a GATA binding site in a regulatory region upstream of the platelet glycoprotein gene causes Gilbert’s syndrome [5]. More recently, human melanoma studies have revealed highly recurrent mutations in the TERT promoter, potentially impacting regulatory elements [6-8].

The emergence of whole-genome sequencing studies in cancer has highlighted the importance of analyzing mutations lying within *cis*-regulatory elements [6,7,9-13]. However, the global relationship between somatic nucleotide variations and the creation or disruption of TFBSs impacting gene expression in cancer is largely unknown [14].

Several attempts have been made to predict the degree to which mutations disrupt TFBSs with TF binding profiles (that is, position weight matrices, PWMs), population genetics, phylogenetic footprinting and experimental data (DNase-seq, epigenetic, etc.) [12,13,15-19]. Mutations at critical positions of TF binding profiles (corresponding to high information content) are the most deleterious for TF–DNA binding [17], thus modelling impact by PWMs is an effective strategy for predicting the impact of a mutation [20]. However, mutations at the more variable, low information content positions of TFBSs can also be functionally constrained [17,21]. Furthermore, relating a TFBS to the gene(s) it regulates presents additional challenges in predicting *cis*-regulatory mutations impacting gene expression. A common simplifying assumption is that a TFBS regulates its closest gene. This first approximation does not consider distal regulation; however, recent analyses of chromatin immunoprecipitation coupled to high-throughput sequencing data sets [22] (the so-called chromatin immunoprecipitation sequencing or ChIP-seq procedure) showed there was accurate prediction of TF gene targets using this approach [23]. In this study, we propose that direct measurements of *cis*-regulatory mutations and gene expression in the same tumor samples will optimally identify mutations in TFBSs impacting the gene expression program in cancer cells.

Ultimately, interpretation of mutations in *cis*-regulatory regions of the genome requires accurate annotation of TFBSs. We have taken the approach of coupling experimental data to targeted computational analysis with TF binding profiles. The ENCODE project [24] and other independent analyses provide a rich resource for locating the key regulatory positions by providing genomic regions bound by TFs derived from ChIP-seq data sets. This provides unprecedented means by which to investigate altered TFBSs and gene regulation in cancer samples [12]. Together with matched gene expression profiles, analysis of mutations in well-annotated TFBS lying in ChIP-seq regions provides a robust set of complementary measurements to study the characteristics of dysregulation through mutation of *cis*-regulatory elements.

We set out to characterize the impact of *cis*-regulatory somatic mutations on gene expression. We focused on two cohorts of patients with B-cell lymphomas (BCLs) [25,26], for which 84 trios (the cancer genomes, matched patient normal genomes and RNA expression from RNA-sequencing (RNA-seq)) were analyzed. We identified cancer-specific somatic mutations across the genome, considering single nucleotide variants (SNVs) and small insertions and deletions (indels) and centered our analysis on *cis*-regulatory elements corresponding to TFBSs predicted within TF-bound regions delineated as ChIP-seq peaks. The regulatory space defined in this analysis by predicted TFBSs within ChIP’ed regions covered approximately 2% of the human genome. We analyzed the location of mutations overlapping TFBSs and revealed that they frequently target promoter regions of apoptotic genes. Integrative analysis of the mutations and gene expression data from RNA-seq highlighted candidate regulatory-disrupting variations as potentially altering expression of genes involved in cancer development. Mutations in *cis*-regulatory elements were frequent, and high-quality candidates in the regulatory set were observed to target genes mutated in the coding space in other samples. We conclude that analysis and interpretation of the *cis*-regulatory genome of cancers will meaningfully augment biological discovery in future studies, resulting in novel mechanistic insight into the genesis malignant phenotypes.

## Results

**Figure 1:**
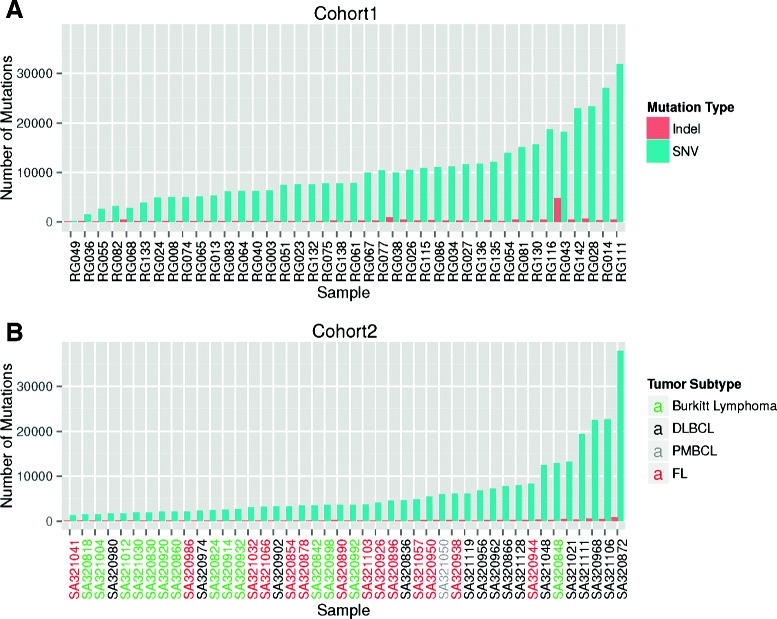
Distribution of the number of mutations per sample in cohorts 1 and 2.**(A)** Number of SNVs (blue) and indels (red) on the *y*-axis are given for all the samples in cohort 1 on the *x*-axis. The samples are ordered from the least number of mutations (left) to the most (right). **(B)** The same type of distribution for the samples in cohort 2. Sample names on the *x*-axis are color-coded by tumor subtype: Burkitt lymphomas (green), diffuse large B-cell lymphomas (DLBCLs, black), primary mediastinal large B-cell lymphomas (PMBCLs, gray) and follicular lymphomas (FLs, red). The same *y*-axis scale has been used for **(A)** and **(B)** for comparison. DLBCL, diffuse large B-cell lymphoma; FL, follicular lymphoma; PMBCL, primary mediastinal large B-cell lymphoma; SNV, single nucleotide variant.

We analyzed somatic mutations extracted from whole-genome sequencing of 84 BCL samples along with matching normal samples from the same individuals. The full set of samples is composed of 40 diffuse large B-cell lymphomas (DLBCLs) (cohort 1) and 44 patients of mixed histology (cohort 2: 14 Burkitt lymphomas, 15 DLBCLs, 1 primary mediastinal large B-cell lymphoma (PMBCL) and 14 follicular lymphomas). RNA expression profiling data (from RNA-seq) were also available for each of the 84 cancer samples plus 62 additional lymphoma samples (52 associated with cohort 1 and 10 with cohort 2). SNV and indel analyses of the data sets from the two cohorts were performed independently as data were derived from different sequencing methods. Somatic mutations in cohort 1 were identified with MutationSeq [27], whereas mutations from cohort 2 were retrieved from the original publication [26]. In aggregate, we observed 406,611 SNVs (from 146 to 31,874 per sample; mean=10,165, median=7,821 and standard deviation (sd)=6,995) and 15,739 indels (from 65 to 4,810 per sample; mean=393, median=222 and sd=735) in samples from cohort 1 and 282,636 SNVs (from 1,242 to 37,987 per sample; mean=6,424, median=3,577 and sd=7,165), and 8,080 indels (from 67 to 871 per sample; mean=184, median=136 and sd=142) in samples from cohort 2 (Figure 1). The distribution of mutations and mutation types over the samples followed a similar pattern in the data sets from the two cohorts including the maximum number of mutations, >30,000 (Figure 1 and Additional file 1: Figure S1). Histological types from cohort 2 clustered by the number of mutations. Namely, Burkitt lymphomas harbored fewer mutations than follicular lymphomas, while DLBCLs harbored the highest number of mutations, consistent with the number of mutations observed within cohort 1 (Figure 1).

### Defined *cis*-regulatory elements showed a higher mutation rate than protein-coding exons but were less mutated than their flanking regions

We began by first identifying mutations lying within *cis*-regulatory elements. We considered TFBSs to be *cis*-regulatory elements and mutations overlapping TFBSs were assumed to be *cis*-regulatory mutations. TFBSs were predicted within ChIP-seq peak regions, collected from multiple cell types and tissues, at whole-genome scale using TFBS profiles from the JASPAR database [28] (see ‘Materials and methods’). We used 477 ChIP-seq data sets (collected for the last update of the JASPAR database [28]) to predict TFBSs associated with 103 TFs (107 JASPAR profiles). Predicted TFBSs covered 76,160,599 bp of the human genome in our analysis. As expected, we observed a very strong enrichment for predicted TFBSs in promoter regions of protein-coding genes with >5 times more nucleotides covered by TFBSs than expected by chance (11,073,418 bp from TFBSs overlapping promoters covering 85,296,239 bp). The portion of the genome covered by predicted TFBSs represents approximately 2% of the chromosomes. We noted the *cis*-regulatory space, which only overlapped the protein-coding space by 6%, covered a proportion of the human genome similar to protein-coding exons.

**Figure 2:**
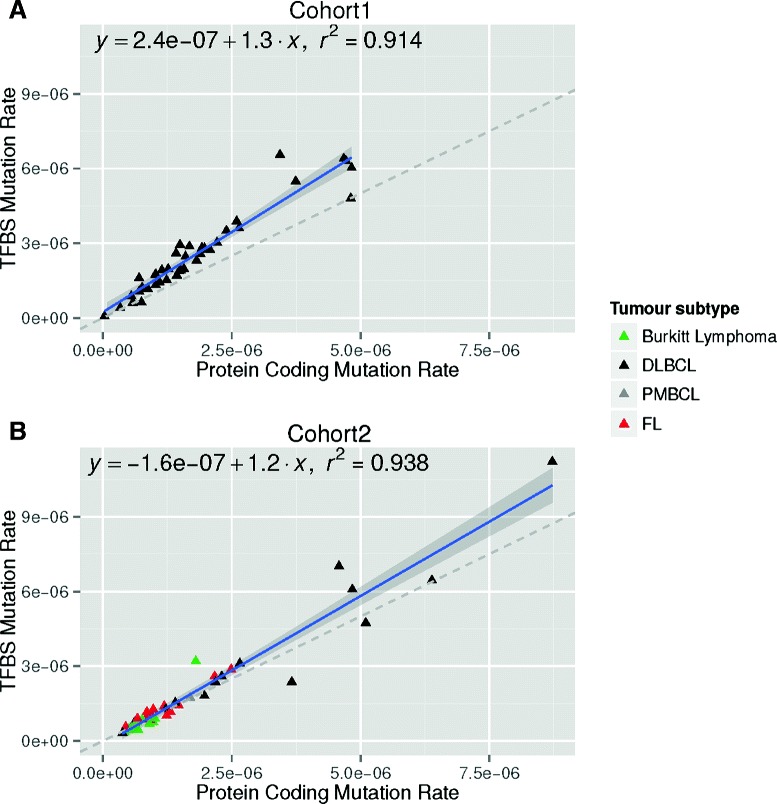
Comparison of the mutation rates in the *cis*-regulatory and protein-coding spaces. Only SNVs from cohort 1 **(A)** and cohort 2 **(B)** have been considered (see Additional file 1: Figure S2 for indels). TFBS mutation rates (*y*-axis) and protein-coding mutation rates (*x*-axis) are plotted for all the samples in cohort 1 **(A)** and cohort 2 **(B)**. Each triangle represents a sample and is color-coded depending on the tumor subtype as in Figure 1. Dashed gray lines represent the identity function (*x*=*y*). Blue lines represent the linear regressions computed from the samples in the two data sets. The equations corresponding to the linear regressions (*y*∼*x*) are written on top of the plots along with the computed *r* ^2^ statistical measures. Dark gray areas surrounding the blue lines provide the 95% confidence region. The same *x*- and *y*-axis scales have been used for both cohort 1 **(A)** and cohort 2 **(B)**. DLBCL, diffuse large B-cell lymphoma; FL, follicular lymphoma; PMBCL, primary mediastinal large B-cell lymphoma; SNV, single nucleotide variant; TFBS, transcription factor binding site.

From the 422,350 mutations predicted from cohort 1, 8,184 (approximately 2%) overlapped TFBSs. Likewise, 6,608 of 290,716 mutations (approximately 2%) overlapped TFBSs in cohort 2. By comparison, 4,990 mutations (approximately 1%) and 5,098 mutations (approximately 2%) overlapped protein-coding exons in cohorts 1 and 2, respectively. SNV mutation rates were higher in TFBSs than protein-coding exons for 38 (95%) cohort 1 and 25 (57%) cohort 2 samples (Figure 2). Analysis of 1,000 simulated genomes with a random mutation distribution shows TFBSs with a higher mutation rate than exons is expected by chance for 540 (respectively, 616) samples in cohort 1 (respectively, cohort 2). The majority of DLBCL (10 of 15) and follicular lymphoma (9 of 14) cohort 2 samples showed a higher mutation rate in TFBSs than in exons; however, the reverse was observed for Burkitt lymphomas (5 of 14) (Figure 2B). Indel mutation rates were similar in TFBSs and exons (Additional file 1: Figure S2).

**Figure 3:**
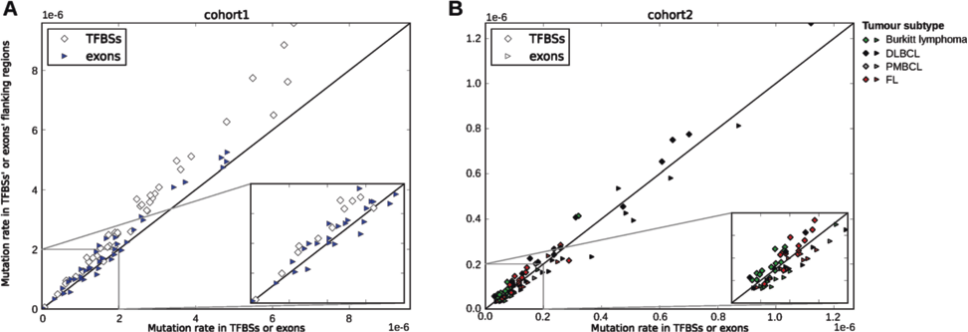
Local SNV mutation rates for TFBSs and protein-coding exons. Mutation rates are plotted for TFBSs and exons (*x*-axis) versus their 1-kb flanking regions on both sides (*y*-axis). Each sample from cohort 1 **(A)** and cohort 2 **(B)** is represented by a square for mutation rates in TFBSs (and their flanking regions) and a triangle for mutation rates in exons (and their flanking regions). Tumor subtypes are color-coded (see legend) like in Figure 1. Results are for SNVs. Figures corresponding to local indel mutation rates are provided in Additional file 1: Figure S3. DLBCL, diffuse large B-cell lymphoma; FL, follicular lymphoma; PMBCL, primary mediastinal large B-cell lymphoma; SNV, single nucleotide variant; TFBS, transcription factor binding site.

TFBSs were less mutated than their flanking regions in both cohorts (Figure 3). Namely, 38/40 cohort 1 and 37/44 cohort 2 samples exhibited lower SNV mutation rates in TFBSs compared to flanking regions. In contrast, only 30/40 cohort 1 and 3/44 cohort 2 samples showed lower SNV mutation rates in protein-coding exons compared to flanking regions (Figure 3). The difference observed for cohort 2 is consistent with the above stated comparison of TFBS and exon SNV mutation rates. Local mutation rates in TFBSs and exons were found to be similar to their flanking regions for indels (Additional file 1: Figure S3). Taken together, these results indicate that predicted TFBSs have lower SNV mutation rates than their flanking regions in both cohorts, while indels are more randomly distributed.

### Promoters of apoptotic genes are frequently targeted regions for *cis*-regulatory mutations

We further explored the impact of *cis*-regulatory mutations by investigating their distribution along the human genome. We sought to characterize the accumulation of mutations in TFBSs lying within the promoters of genes implicated in pathways known to be disrupted in cancer development. We quantified mutation rates in 1-kb-long sliding windows across the genome, identifying windows where at least three mutations were found (the two cohorts were analyzed independently and combined).

**Figure 4:**
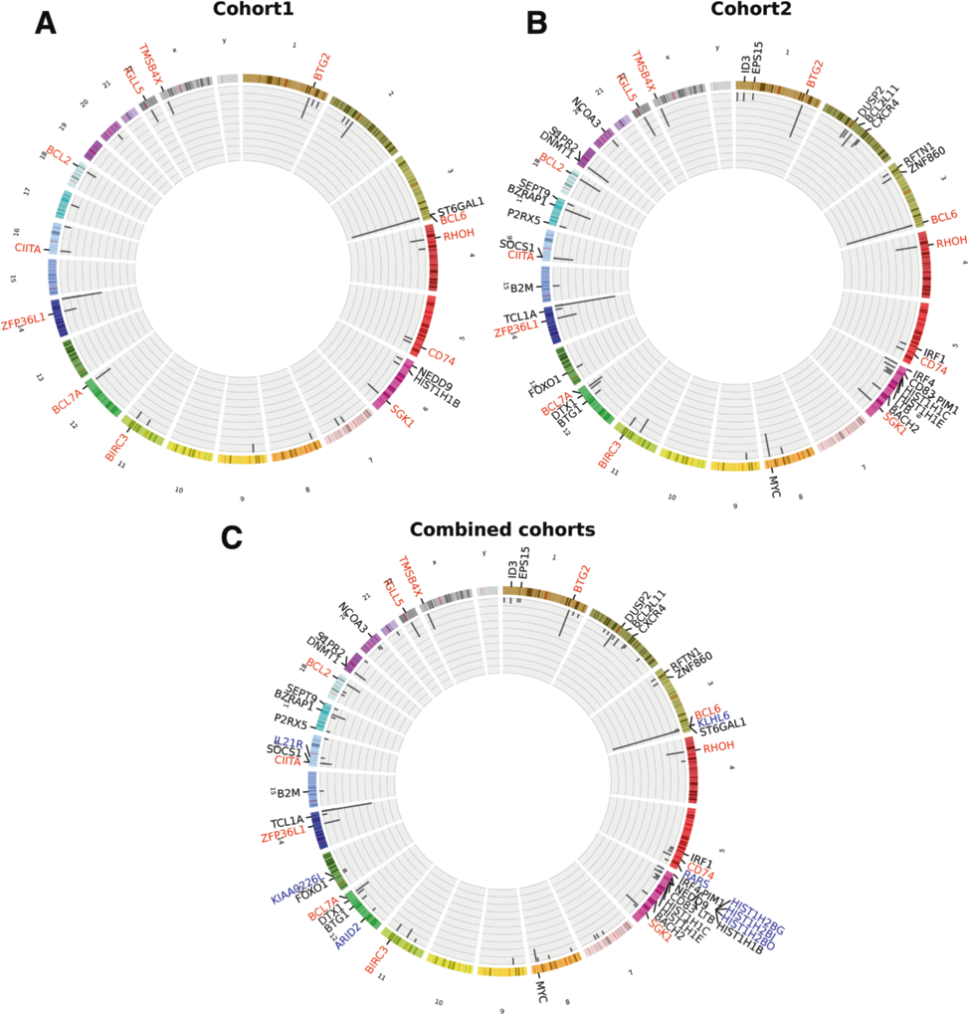
Regions frequently targeted by somatic mutations overlapping *cis*-regulatory elements are enriched in promoters. A 1-kb-long window was slid with a 500-bp step along the human chromosomes and we recorded the number of overlapping mutations at each position. The corresponding histogram is given in the inner gray circles of the Circos plots where only positions containing at least three mutations have been retained. The *y*-axis range for the histograms is [0, 40] for **(A)** and **(B)** and [0, 80] for **(C)**. The outer circles contain the names of the genes closest to the mutations in the considered windows if the mutation is at most 2 kb away from the TSS of the gene. Names highlighted in red correspond to genes shared between the analyses for the cohort 1 **(A)** and the cohort 2 **(B)** data sets. Genes highlighted in blue are specific to the analysis of the mutations when combining somatic mutations from the two cohorts. Analyses have been applied to the set of mutations from cohort 1 **(A)**, cohort 2 **(B)** and both cohorts combined **(C)**. TSS, transcription start site.

Frequently mutated regions are significantly enriched for promoters of protein-coding genes (Figure 4). Namely, 135 mutations in frequently targeted regions were ≤2 kb away from a protein-coding gene’s transcription start site (TSS) using the samples from cohort 1 (representing approximately 49% of all 273 mutations found in frequently targeted regions, *P*=1.16×10^−75^, hypergeometric test with 680 mutations overlapping TFBSs in promoters out of 8,185 in TFBSs). In cohort 2 samples, 348 mutations in frequently targeted regions were within promoters (approximately 65% of the 534 found in frequently targeted regions, *P*=3.28×10^−156^, hypergeometric test with 1,102 mutations overlapping TFBSs in promoters out of 6,608 in TFBSs). We compiled the set of mutations found in the frequently targeted regions within promoters and extracted the closest protein-coding gene to each mutation. Twelve genes were frequently targeted in both cohorts independently (Figure 4A,B), including BCL2, BCL6, BCL7A, CD74 and CIITA, all listed as oncogenes in the Cancer Gene Census [29] and known to be involved in lymphomagenesis. An additional 13 genes (ARID2, BCL2L11, BZRAP1, EPS15, HIST1H2BG, ID3, IGLL5, IL2R1, IRF1, KIAA0226L, NEDD9, RARS and ZNF860) from combined cohort analysis (Figure 4C) had not been previously described as aberrant somatic hypermutated regions [30]. Six of these genes (ARID2, BCL2L11, EPS15, IL2R1, NEDD9 and ZNF860) were exclusively mutated in their promoters (that is, no mutations in exons were observed). Our data indicated for the first time that ID3 (recurrently mutated in Burkitt lymphomas [26]) can be targeted through TFBS mutations in its promoter region. Thus, both exonic and promoter portions of the gene are recurrently mutated, suggesting complementary genetic mechanisms for gene disruption.

Mutations in frequently targeted regions overlapped five enhancers in cohort 1 and five enhancers in cohort 2 (there were two enhancers in common: intronic enhancers of BIRC3 and ST6GAL1). Four of the enhancers targeted in cohort 1 are intronic enhancers for the genes BCL2, BCL7A, BIRC3 and ST6GAL1 while the fifth is located in the intergenic region between BCL6 and LPP. All five enhancers found in cohort 2 are intronic enhancers in genes BCL2, BIRC3, CIITA, IGLL5 and ST6GAL1. All these genes have already been associated with hypermutated regions in BCLs (BCL2, BCL6, BCL7A, BIRC3, CIITA and ST6GAL1) [30] or listed in the Cancer Gene Census (BCL2, BCL6, BCL7A, BIRC3, CIITA, IGLL5 – in the IGL@ locus – and LPP).

**Figure 5:**
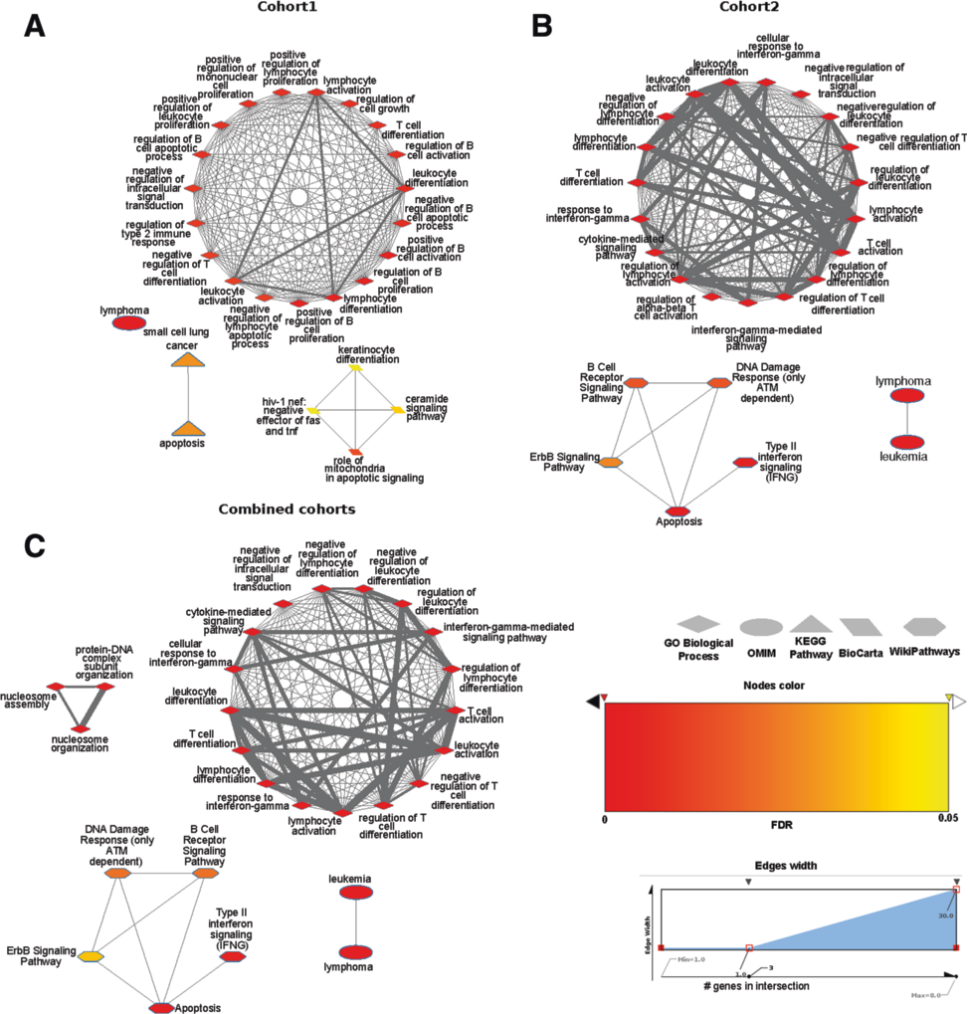
Functional enrichment analyses of genes associated with frequently mutated regions. Enrichr [31] functional enrichment analyses were realized for the sets of genes listed in Figure 4 for cohort 1 **(A)**, cohort 2 **(B)** and the two data sets combined **(C)** (see Additional file 2). The enriched pathways with the 20 lowest Bonferroni corrected *P* values are shown for each category only where Bonferroni corrected *P*<0.05 (see Additional file 2 for the complete Enrichr results). Each node of the graphs represents an enriched pathway where the color of a node represents its Bonferroni corrected *P* value. An edge between two nodes indicates that the pathways share genes. The larger the width of the edge, the larger the number of shared genes. FDR, false discovery rate; GO, gene ontology; OMIM, online mendelian inheritance in man.

To synthesize our observations from the gene level, we next analyzed genes with frequently targeted promoters through pathway enrichment analysis. All the genes highlighted in Figure 4 were submitted to Enrichr [31]. Both cohorts were analyzed separately (genes from Figure 4A,B) and combined (genes from Figure 4C). We identified enrichment (adjusted *P*<0.05) for apoptotic processes (Figure 5 and Additional file 2) including apoptosis, regulation of the B-cell apoptotic process and cell-type-specific apoptotic processes. The genes associated with the apoptotic terms are BCL2, BCL2L11, BIRC3, BTG1, CD74, IRF1, IRF4 and MYC. Moreover, we observed enrichment for B-cell and oncogenic related pathways (for example, the B-cell receptor signaling pathway, small cell lung cancer, regulation of B-cell proliferation, lymphoma and leukemia) as shown in Figure 5 and Additional file 2. Taken together, these results highlight that apoptotic genes, and oncogenic processes in general, are frequently targeted by mutations within TFBSs found at their promoter regions.

### Landscape of *cis*-regulatory mutations impacting gene expression in B-cell lymphomas

We next assessed the impact of *cis*-regulatory mutations on gene expression. We used a novel probabilistic model, called xseq [32], to relate specific mutations to expression disruption in pathways (see ‘Materials and methods’). The approach assesses the likely association of the presence of mutations with observed deviations from neutral expression measurements taken from the same tumor. The method takes as input a patient-gene expression matrix and a binary patient-gene mutation matrix and outputs the probabilities that: (a) a mutated gene (over the whole patient population) impacts gene expression and (b) a patient-specific mutated gene impacts expression in the patient. xseq was originally developed for genes harboring mutations in their protein-coding exons only. Here, we extended xseq to highlight *cis*-regulatory mutations potentially deregulating transcriptional regulation. We encoded a gene as mutated in the patient-gene mutation matrix when it was the closest gene to a *cis*-regulatory mutation and it was up- or down-regulated in the mutated sample compared to other samples. With the applied criteria, a TFBS was associated with a single gene but a gene might be associated with several TFBSs. To provide xseq with a complete view of mutated genes, we incorporated both genes mutated in their protein-coding regions and genes showing altered expression associated with mutations in their regulatory regions (Additional file 1: Figure S4 and ‘Materials and methods’). By combining these tagged genes with expression data from RNA-seq, we used xseq to predict candidate mutated genes associated with altered expression and linked to genes in biological networks harboring altered expression in the same samples.

**Figure 6:**
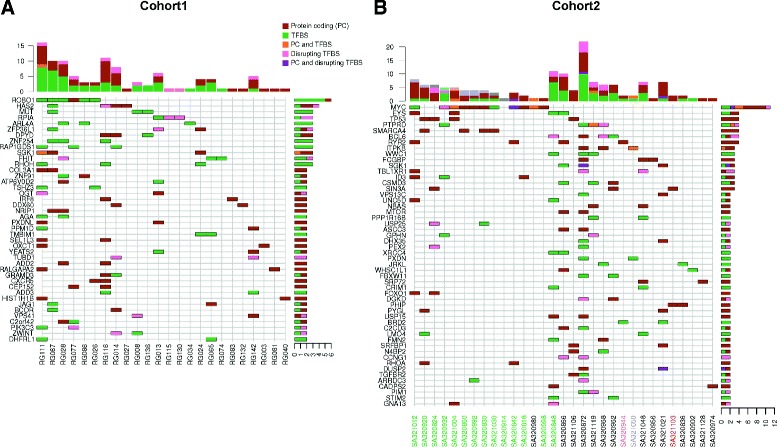
xseq results. Cancer genes predicted by the xseq tool from the cohort 1 **(A)** and cohort 2 **(B)** data sets. Each row corresponds to a predicted gene and each column to a cancer sample. When a gene is predicted in a specific sample, a colored box is drawn. Box colors indicate the type of mutation associated with the gene (in protein-coding exons only, brown; in TFBS only, green; in protein-coding exon and TFBS, orange; in a TFBS only and predicted to disrupt the TFBS, pink; and in protein-coding exon and TFBS and predicted to disrupt the TFBS, purple). The histograms on the right sum the number of samples where the gene is predicted (using the same box colors). The histograms at the top sum the number of genes predicted by xseq in samples (using the same box colors). Cohort 2 sample names **(B)** are color-coded as defined in Figure 1. PC, protein coding; TFBS, transcription factor binding site.

**Figure 7:**
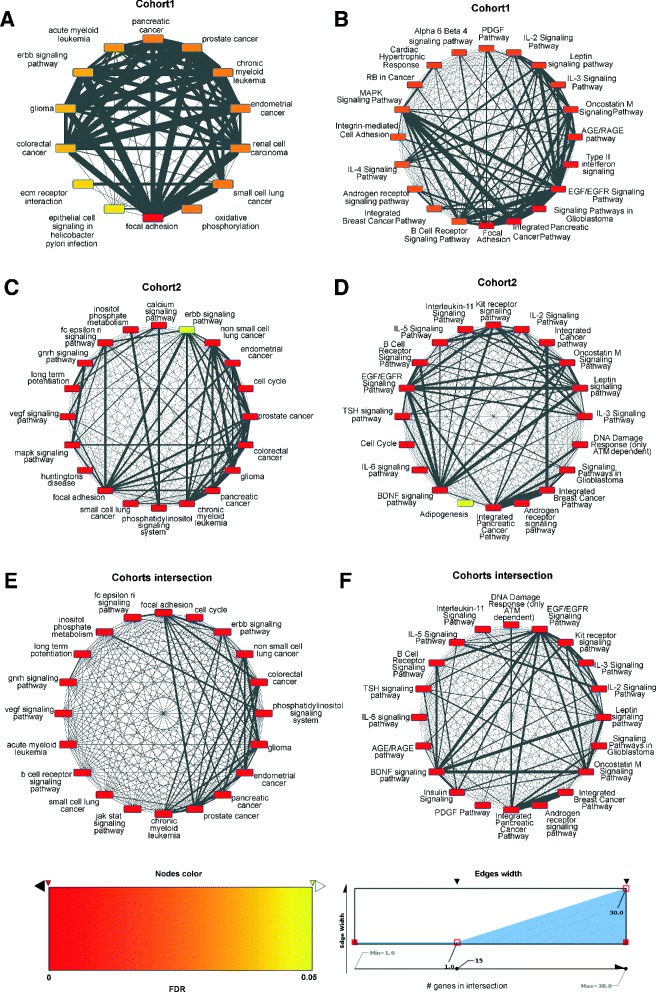
Functional enrichment analyses of disrupted pathways. xseq-predicted genes along with their neighbors in biological pathways showing altered expression were derived from the xseq analyses (see ‘Materials and methods’ and Additional file 2). Functional enrichment was performed with Enrichr [31] using the genes obtained from the cohort 1 **(A,B)** and cohort 2 **(C,D)** data sets and their intersection **(E,F)** (see Additional file 2). The enriched terms from KEGG **(A,C,E)** and WikiPathways **(B,D,F)** with the 20 lowest Bonferroni adjusted *P* values are shown. Only terms with a Bonferroni corrected *P*<0.05 are conserved (see Additional file 2 for the complete Enrichr results). Each node of the graphs represents an enriched pathway where the color of a node represents its Bonferroni corrected *P* value. An edge between two nodes indicates that the pathways share genes. The larger the width of the edge, the larger the number of shared genes. FDR, false discovery rate.

A total of 42 genes were predicted in cohort 1 samples along with 5,412 biological network neighbors with altered expression (Figure 6A). The same analysis applied to cohort 2 samples led to 1,533 deregulated biological network genes connected to the 52 xseq-predicted genes with altered expression associated with mutations (Figure 6B). The sets of genes captured by xseq along with their deregulated neighbors were enriched for pathways related to cancer and cancer development (Figure 7A,B,C,D and Additional file 2). The sets of 5,554 genes from cohort 1 and 1,585 genes from cohort 2 had an intersection of 829 genes (Additional file 2). Functional enrichment analysis highlighted strong over-representation of cancer-related genes (Figure 7E,F and Additional file 2), reinforcing the predictions from xseq as being involved in cancer development *a posteriori*. Note that four genes in cohort 1 (HIST1H1B, RHOH, SGK1 and ZFP36L1) and seven in cohort 2 (BCL6, DUSP2, ID3, FOXO1, MYC, PIM1 and SGK1) were associated with frequently targeted promoters (Figure 4) and predicted by xseq (Figure 6).

Ranking the predicted genes by the number of samples in which they were dysregulated highlighted known cancer driver genes such as MYC, TP53, ID3 and BCL6 (Figure 6). Burkitt lymphomas tended to be segregated from the other types of BCLs where MYC was predicted as a mutated gene with altered expression. MYC was predicted by xseq for 11 samples, 10 of which are Burkitt lymphomas. In all of the 11 samples, MYC up-regulation was observed (Additional file 1: Figure S5), in agreement with the oncogene function of MYC in cancers [33].

We next characterized the distribution of mutations in genes impacting gene expression in protein-coding and *cis*-regulatory regions. We categorized each gene as associated with: (1) a protein-coding mutation, (2) a *cis*-regulatory mutation or (3) both (Figure 6). Some genes were predicted with altered expression and associated with mutations in their exons only (for example, TP53, RYR2 and SIN3A in cohort 2 and COL3A1, IRF8 and NRIP1 in cohort 1). We also observed multiple genes predicted in multiple samples consistent with alternate mechanisms of alteration. For instance, HAS2 and ZFP36L1 in the cohort 1 data set and MYC and BCL6 in the cohort 2 data set were associated with mutations either in the *cis*-regulatory or the protein-coding spaces. For ID3, gene expression alteration was associated with a mutation in a TFBS in the SA320932 sample whereas it was associated with mutations in the exons in the two other samples (SA321012 and SA320818) (Figure 6B).

### Examples of genes with *cis*-regulatory mutations associated with expression dysregulation

**Figure 8:**
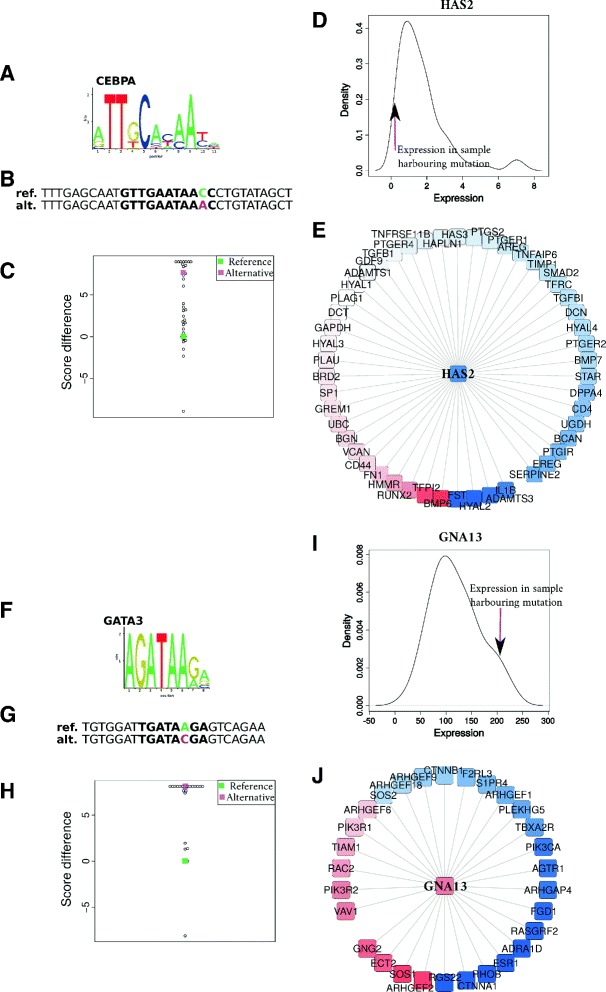
Examples of predicted *cis*-regulatory mutations potentially impacting gene expression. HAS2 **(A,B,C,D,E)** and GNA13 **(F,G,H,I,J)** have been predicted by xseq for samples RG116 and SA320848, respectively. In RG116, a CEBPA TFBS (TF binding profile in **(A)**) is predicted to be disrupted (see reference and alternative sequences in **(B)** where the SNV is highlighted with the reference nucleotide in green and the alternative in purple). Score differences between the reference TFBS and all possible alternative TFBSs are plotted in **(C)**. The distribution of HAS2 expression from RNA-seq data is plotted in **(D)** with an arrow pointing to the expression value in sample RG116. **(E)** represents the network of genes associated with HAS2, which are predicted to be either down- (blue) or up-regulated (red) in RG116. The higher the opacity, the stronger the down- or up-regulation. Similar plots are given in **(F,G,H,I,J)** for GNA13 in SA320848 with a potentially disrupted GATA3 TFBS. alt., alternative; ref., reference; SNV, single nucleotide variant.

xseq analyses highlighted the specific mutations associated with gene-expression dysregulation along with cascading effects on interactors through functional protein association networks. For instance, specific SNVs were predicted as deleterious for TFBSs and associated with expression dysregulation of the genes HAS2 and GNA13 (Figure 8 and Additional file 3). Recurrent dysregulation associated with *cis*-regulatory mutations was also observed as exemplified in the promoter of BCL6 along with a potential cascading effect on interactors known to be involved in cancer development (Additional file 1: Figures S7 and S8 and Additional file 3). As a last example, our approach highlighted SNVs in TFBSs associated with the promoters of ROBO1 for five DLBCL samples (Figure 6 and Additional file 1: Figure S9). ROBO1 was down-regulated in all of these five samples (Additional file 1: Figure S10). We hypothesize that ROBO1 is a tumor suppressor (as suggested in [34-36]), whose dysregulation shows recurrent altered expression of its interactors SOS1, SOS2 and RAC1, which are associated with carcinogenesis [36,37] (Additional file 1: Figure S10 and Additional file 3). These observations shed light on the supposed tumor suppressor role of the ROBO1 gene. We highlight that ROBO1 might be down-regulated in some DLBCLs at the transcriptional level by *cis*-regulatory mutations since no mutations were found in the protein-coding space in these samples.

## Discussion

Our results reveal the importance of fully characterizing somatic mutations in *cis*-regulatory regions of cancer genomes. Whole-genome sequencing data from lymphoma samples indicated somatic mutations impacting TFBSs and events associated with alteration in transcription. These results demonstrated that interpretation of mutations in cancer genomes will be substantially enhanced by consideration of mutations impacting *cis*-regulatory regions and with joint analysis of gene expression data acquired from the same tumor tissue.

We expect our results to be an underestimate of the functional non-coding mutational landscape. Our approach relied on high-quality annotations of the *cis*-regulatory space, capitalizing on the availability of a large volume of experimentally derived TF-DNA interactions from ChIP-sequencing. We used manually curated TF binding profiles from the JASPAR database to predict TFBSs within regions bound by ChIP’ed TFs. Although we combine ChIP-seq experiments from multiple cell types and conditions, the experimental ChIP-seq information provides the best current opportunity to focus on the non-coding space. The set of predicted TFBSs covered approximately 2% of the human genome, a similar proportion to the coding regions, and harbored lower SNV mutation rates than their surrounding regions. However, we can expect that the robustly annotated regulatory space of the human genome will grow over the coming years with the availability of more antibodies, decreasing costs and broader coverage of cell types. As such, it is likely that additional *cis*-regulatory regions will be found aberrant in tumor genomes, allowing for more comprehensive interpretation of genome-wide somatic mutations driving malignant phenotypes.

Detection of genes with altered expression due to the disruption of regulatory TFBSs is the subject of ongoing research. There is a need for better prediction of the impact of mutations on TF–DNA binding affinity. Multiple approaches have been explored over the years to tackle this problem by considering the score difference between reference and alternative sites [15] or the decrease of the reference binding compared to the alternative binding score [12]. Here, we considered all mutations lying within TFBSs of potential interest, highlighting the ones that are the most likely to disrupt TFBSs where the alternative score was below a defined threshold. We suggest this approach is simple and conservative. Therefore, future improvements are likely to increase sensitivity when coupling ChIP-seq data to TFBS variant prediction.

The prediction of TFBSs within ChIP-seq peaks is performed without considering the competing environment between TFs with different specificities. For instance, the down-regulation of BCL6 in SA320962 is associated with a mutation not predicted to disrupt the STAT3 TFBS while STAT3 is known as an activator. A hypothesis is that competition between STAT3 and STAT5 occurs at this binding site, since they recognize similar motifs and a previous study highlighted that STAT5 outcompetes STAT3 for repressing the expression of BCL6 [38]. The mutation might then provide an advantage for STAT5 binding at this location. Functional studies based on our analyses would be required to decipher the mechanisms underlying the competition between TFs at TFBS loci to understand further the impact of mutations on gene regulation.

We aimed to predict the mutations most likely to have an impact on gene expression through the disruption of regulatory elements (TFBSs). The approach focused on gene expression alteration was built on top of the protein-coding changes to provide new insights into gene expression dysregulation of cancer driver genes. We showed that some genes have been predicted in multiple samples using different mechanisms of dysregulation through either protein-coding or TFBS alterations. We took a naive approach in this study to associate mutations/*cis*-regulatory elements with a gene. This approach is relevant when looking at promoter regions but we will ultimately require more information about the association of distal regulatory elements with promoters. Cell-specific (or cell-type-specific) experimental profile comparisons and expansion of chromatin conformation capture data sets will empower analysis linking distal regulatory elements to their targets.

With the forthcoming availability of cancer whole-genome sequence data coupled with gene expression data at large scale, the analysis of non-coding *cis*-regulatory elements will be critical for understanding cancer. Our results indicated this will be fruitful, yielding additional cancer biology to aid in closing the discovery gap in large-scale studies that have focused exclusively on the protein-coding component of the genome. We suggest that combining the impact of mutations on transcriptional regulation, protein products and post-transcriptional regulation at genome scales will empower comprehensive biological interpretation of human malignancy.

## Conclusions

In this report, we analyzed a set of approximately 700,000 somatic SNVs and indels in 84 BCL samples to provide an initial genome-scale foray into the analysis of *cis*-regulatory mutations impacting gene expression in cancer. By overlapping the somatic mutations with predicted TFBSs within ChIP-seq regions, we looked at the distribution of mutations overlapping TFBSs in the human genome. We highlighted that *cis*-regulatory mutations are frequently situated in promoters. The set of genes with promoters targeted by mutations within TFBSs are enriched for apoptosis-related and carcinogenesis pathways. Finally, by combining mutation information with gene expression from RNA-seq data, we predicted cancer genes with altered expression associated with mutations found either in exons or in TFBSs associated with the genes. The approach revealed samples where genes were potentially dysregulated through the disruption of *cis*-regulatory elements and highlighted the importance of interrogating the *cis*-regulatory genomic space for somatic mutations in cancer.

## Materials and methods

### Transcription start site, exon and enhancer coordinates

TSS and exonic positions of protein-coding genes (transcript accessors starting with NM_) have been retrieved from the UCSC hg19 Table Browser [39] by selecting the knownGene table from the RefSeq gene track. Enhancer coordinates were retrieved from [40].

### Cancer genome data

The raw sequencing data for the DLBCL data set of cohort 1 were retrieved from [25]. We obtained the corresponding RNA-seq data from the same publication. The already processed sets of mutations and RNA-seq expression level data for cohort 2 [26] were retrieved from the ICGC data portal [41].

### Expression data computation

Expression data for samples in cohort 1 were processed using the *Rsamtools* and *GenomicFeature* [42] Bioconductor [43] packages to generate gene expression levels from the RNA-seq raw data. We only considered genes with an official HGNC symbol [44]. Finally, genes with null expression over all the samples were filtered out. The final set of official HGNC symbols for the considered genes can be found in Additional file 4. We did not consider copy number alteration information for both cohorts since the data were not available for cohort 2.

### Single nucleotide variant predictions

SNVs were identified for cohort 1 samples using a modified version of MutationSeq [27,45]. We filtered out SNVs with probability <0.9.

### Indel predictions

We used Dindel [46] to call indels in the samples from cohort 1. Dindel identified indels of length 1 to 50 bp. Following Dindel’s manual recommendations, we provided Dindel with the BAM file and the set of candidate indels obtained from Pindel [47] for each sample. Default parameters were used for Dindel. Pindel indel candidates were obtained using the default parameters except the insert size, which was provided by the CollectInsertSizeMetrics Picard subtool [48]. We only considered indels with Dindel quality scores greater than or equal to ten, which is equivalent to 90% confidence. All variants reported in dbSNP (version 132) [49] and the 1,000 genomes project [21] were filtered out. Identifying the specific location of an indel within a homopolymer or tandem repeat is challenging, which effects the ability to label an indel properly as somatic or germline. Therefore, we labeled indels as germline mutations if the distance of the repeating region from the start position of the indel was longer than the distance to the closest indel in the normal sample. The repetitive sequences that were considered can be any combination of base pairs between 1 and 6 bp in length.

### Mutation rates

Mutation rates within TFBSs were computed by dividing the number of SNVs or indels lying within TFBSs by the total number of nucleotides within TFBSs (that is, 76,160,599). The included TFBSs were predicted within ChIP-seq peak regions as described below. A similar computation was performed for protein-coding exons using the exonic start and end positions from RefSeq. The total number of nucleotides covered by the exons is 65,469,364.

When computing local mutation rates, we considered regions directly flanking TFBSs and exons. The flanking regions were obtained using the *flank* and *subtract* subcommands of BEDTools [50] by extracting 1 kb upstream and 1 kb downstream of the TFBSs (respectively, exons) and filtering out sequences overlapping TFBSs (respectively, exons).

Genomes with randomly distributed mutations were constructed by shuffling all the mutations from cohort 1 or cohort 2 in the human genome using the *shuffle* subcommand of BEDTools [50]. Then 1,000 genomes were computed for each cohort. For each genome, we calculated the mutation rates in TFBSs and exons using the randomly positioned mutations.

### ChIP-seq data and transcription factor binding site predictions

We collected 477 human TF ChIP-seq data sets from both ENCODE [24] and publications collected in PAZAR [51] with an associated TF binding profile described in the JASPAR database [28] (Additional file 5). ChIP-seq peak regions called in the studies were retrieved from the corresponding analyses. TF binding profiles for the corresponding 103 TFs were retrieved from the 2014 release of the JASPAR database [28].

TFBS predictions were obtained by scanning PWMs derived from the TF binding profiles (see [52] for the TF binding profile to PWM conversion) using the TFBS Perl module [53]. The PWMs were applied to the whole length ChIP-seq peaks and we further considered in our analysis the hits for which the relative PWM score was over 85% (see [52,53]). The default threshold of 85% was used to call TFBSs as in previous studies [54,55]. We predicted TFBSs covering 76,160,599 bp. Note that both strands on the reference and alternative genomes were scanned with the PWMs to search for the optimal hits.

### Frequently targeted regions

Frequently targeted regions were obtained by sliding a 1-kb window along the human genome with steps of 500 bp. The *makewindows* subcommand of BEDTools [50] was used to construct the set of window coordinates. For each position, we recorded the number of *cis*-regulatory mutations overlapping the window using the BEDTools [50] *intersect* subcommand. Only windows containing at least three mutations were considered and plotted in Figure 4. Mutations from the two cohorts were analyzed separately (Figure 4A,B) and combined (Figure 4C). For each mutation lying within a frequently targeted region, the closest gene was extracted using the TSS positions of the RefSeq genes by applying the *closest* subcommand of BEDTools [50]. Genes with a TSS at a distance of at most 2 kb were used for Figure 4.

### Prediction of alternative transcription factor binding sites

For each mutation, we computed the best PWM score using the alternative sequence containing the mutation. To compute the PWM score, we extracted sequences with a length of 2*n*−1 bp (with *n* being the length of the considered PWM, Additional file 1: Figure S11A) centered around the SNV to identify regions that could contain a better TFBS at any overlapping position on the alternative sequence (Additional file 1: Figure S11B). Alternative TFBS scores resulting from an insertion were computed for sequences of length 2*n*−2+*i* bp where *i* represents the length of the insertion (Additional file 1: Figure S11C). Similarly, alternative TFBS scores resulting from a deletion were obtained by scanning the 2(*n*−1) bp sequence centered at the deletion region (Additional file 1: Figure S11D). When scanning alternative sequences with the PWMs, only the best hit per sequence was recorded. We considered a mutation (SNV or indel) to be deleterious for a TFBS if the PWM relative score was below 80% for the alternative sequence.

### MANTA

All the predicted TFBS positions can be scanned for overlap with SNVs using our dedicated Mongo database for the analysis of TFBS alterations (MANTA). MANTA stores the positions of all predicted TFBSs as well as all the potential SNVs overlapping these positions. For each potential SNV, you can retrieve information about the reference and alternative best TFBSs along with their scores (see ‘Prediction of alternative transcription factor binding sites’ for the computation of the alternative scores). The MANTA source code is available at [56] and the system can be interrogated at [57].

### xseq

xseq analyses were performed as follows.

#### Preprocessing: Identify mutated genes

##### Mutations lying within TFBSs impacting gene expression

Mutations lying within TFBSs were obtained using MANTA. The closest gene to each mutation was obtained using the set of TSSs of known refSeq genes from UCSC. When finding the closest gene, we consider the start and end positions for the mutation and the start positions for all protein-coding TSSs from refSeq. Only mutations lying within TFBSs with a potential impact on gene expression were considered. Namely, we require that the closest gene to the corresponding mutations to be either up- or down-regulated in the sample of interest. To determine if a gene is deregulated, we considered the distribution of expression of the gene in the cancer samples and required that the expression in the corresponding sample was >*μ*+1*σ* or <*μ*−1*σ* where *μ* and *σ* represent the mean and standard deviation of the distribution of expression values.

##### Mutations lying within protein-coding exons

All mutations lying within a protein-coding exon were considered. Namely, SNPeff [58] was used to extract the mutations overlapping protein-coding regions and their predicted impact on the protein. Additional file 6 lists the mutation impacts that were considered in the analysis.

#### xseq analysis

All genes obtained from the previous step were used in the input to the xseq tool, which is a probabilistic model that aims to encode the impact of somatic mutations on gene expression profiles. The model uses a generative hierarchical Bayes approach, which has as input three observed quantities: a patient-gene expression matrix, a patient-gene mutation matrix and a graph containing known interactions between genes (for example, from pathway databases). The model has two key unobserved random variables, which constitute the output: *D*_*g*_ is a Bernoulli random variable where *D*_*g*_=1 indicates that gene *g* influences expression when mutated; ${F_{g}^{p}}|D_{g}$ is a Bernoulli random variable where ${F_{g}^{p}}$ indicates that mutated gene *g* influences expression in patient *p*. As such, we model expression influence at two levels: over the patient population and at the level of individual mutations in individual patients. Random variables are estimated using the belief propagation algorithm, with outputs consisting of two relevant probabilities: Pr(*D*_*g*_) and $\text {Pr}({F_{g}^{p}})$. Software implemented in an *R* package encoding xseq is available at [59,60]. By considering the disruption likelihoods of each mutated gene and its neighbors in biological networks, xseq computes the probability of a mutated gene being deregulated and causing cascading dysregulation effects on its neighbors.

#### Post-processing

xseq provides the probability of each input gene being a driver gene in the specific samples where it is mutated (single-sample probability, $\text {Pr}({F_{g}^{p}})$) as well as its driver potential considering all samples (all-samples probability, Pr(*D*_*g*_)). Potential false positives from xseq are produced when a gene is only mutated in a single sample because there is minimal information for calculating the all-samples probability properly. By plotting a histogram of all the calculated probabilities when considering all samples (Additional file 1: Figure S12), a distinct peak was observed, which is formed by a large number of these false positives. These distinct peaks were used as a threshold; genes must have an all-samples probability greater than or equal to 0.5 in cohort 1 and 0.8 in cohort 2 to be considered in our analyses. Furthermore, we require that the single-sample probability of a gene is greater than or equal to 0.5 and the gene is predicted in at least two samples to be considered in the analysis (Figure 6).

### Circos plots

The Circos plots in Figure 4 were drawn using the Circos tool version 0.64 [61].

### Functional enrichment analyses

Functional enrichment analyses were performed using the Enrichr tool [31] (as of 20 January 2015) through its API using the *poster* library of Python2.7. A term is considered to be enriched if the associated adjusted *P*≤0.05. Visualization for the enrichment plots were constructed manually using Cytoscape 3.1.0 [62]. Enrichment results associated with *Mus musculus* in WikiPathways were filtered out and only *Homo sapiens* associated terms were conserved.

The functional enrichment analysis illustrated in Figure 5 was obtained from the list of genes provided in Figure 4 (Additional file 2). The functional enrichment analysis in Figure 7 was computed using the genes predicted by xseq (Figure 6 and Section ‘xseq’) along with their biological network neighbors with altered expression (that is, predicted by xseq to have a higher probability of being up- or down-regulated than being neutral) in cohort 1, cohort 2 and their intersection (Additional file 2).

### Statistical analyses

Hypergeometrical *P* values were computed using the *phyper* function of the *R* environment [63].

## Additional files

Additional file 1
**Additional figures.** Supplementary figures referenced in the manuscript along with their descriptions.

Additional file 2
**Functional enrichments.** Lists of genes used for the functional enrichment analyses along with the complete Enrichr results with a corrected *P*<0.05.

Additional file 3
**Case examples.** Details of the case examples predicted by xseq (HAS2, GNA13, BCL6 and ROBO1).

Additional file 4
**HGNC symbols.** List of HGNC symbols associated with the genes that were analyzed in this study.

Additional file 5
**ChIP-seq experiments.** List of ChIP-seq experiments used in this analysis along with the associated JASPAR TF binding profiles.

Additional file 6
**Mutation impacts.** List of mutation impacts from SNPeff that have been considered in this study.

## Abbreviations

BCL: B-cell lymphoma
bp: base pair
ChIP-seq: chromatin immunoprecipitation sequencing
kb: kilobase
PMBCL: primary mediastinal large B-cell lymphoma
PWM: position weight matrix
RNA-seq: RNA-sequencing
sd: standard deviation
SNV: single nucleotide variant
TF: transcription factor
TFBS: transcription factor binding site
TSS: transcription start site
