# Supplementary material for: Cis-regulatory somatic mutations and gene-expression alteration in B-cell lymphomas
Source: Genome Biol. 2015 Apr 23;16(1):84. doi: 10.1186/s13059-015-0648-7 (PMC4467049; doi:10.1186/s13059-015-0648-7)
Supplement: Additional file 1 — Additional figures. Supplementary figures referenced in the manuscript along with their descriptions. [file 13059_2015_648_MOESM1_ESM.pdf]

Additional Figures

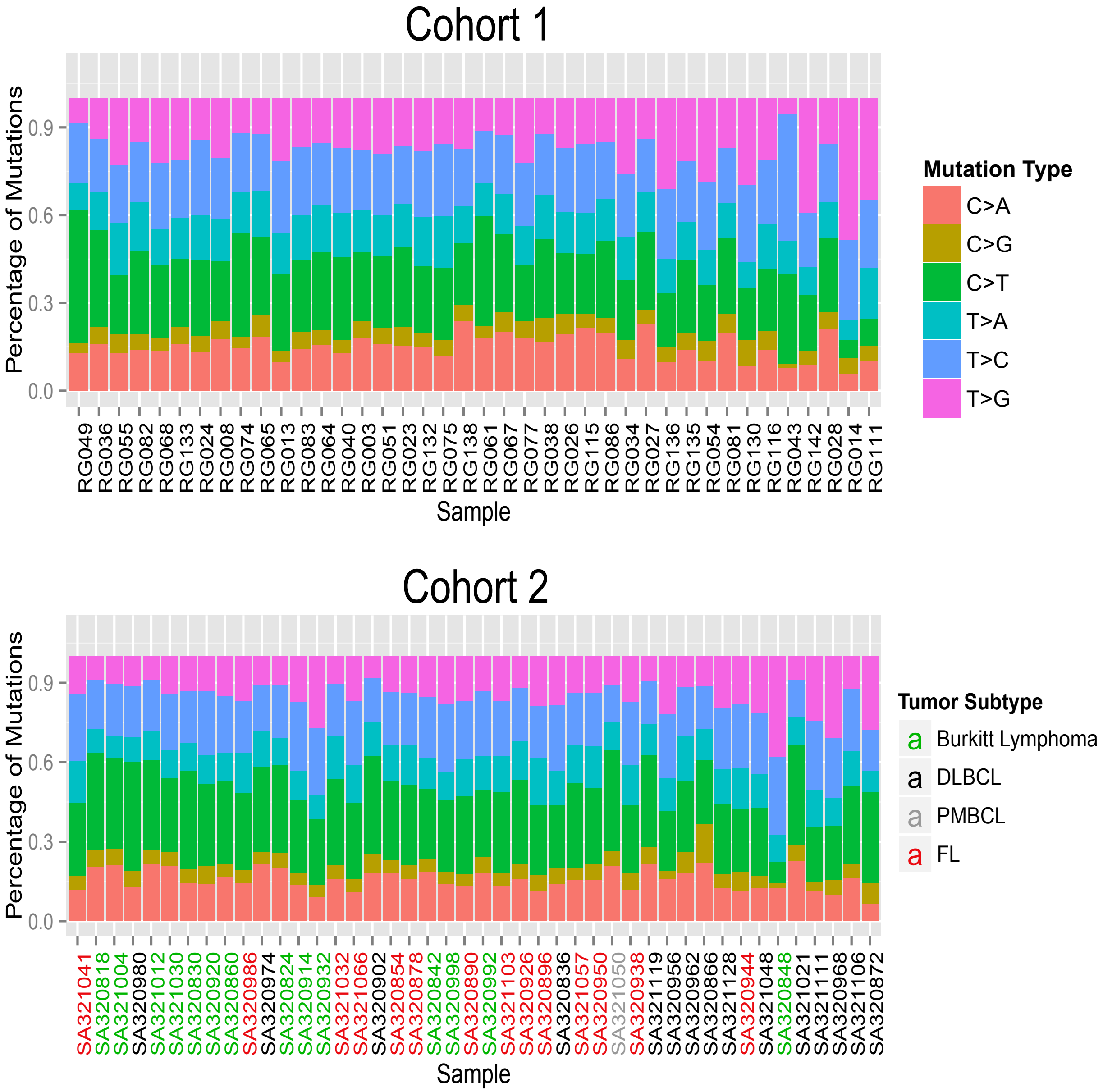

Additional file 1: Figure S1 – Proportion of each SNV type in cohort1 and cohort2 samples. For each sample in cohort1 (**top**) and cohort2 (**bottom**) on the x-axis, we report the proportion of each SNV type (y-axis). SNV types (C>A, C>G, C>T, T>A, T>C, and T>G) are color-coded. Sample names from cohort2 are color-coded per tumor subtype as in Figure 1 in the main manuscript.

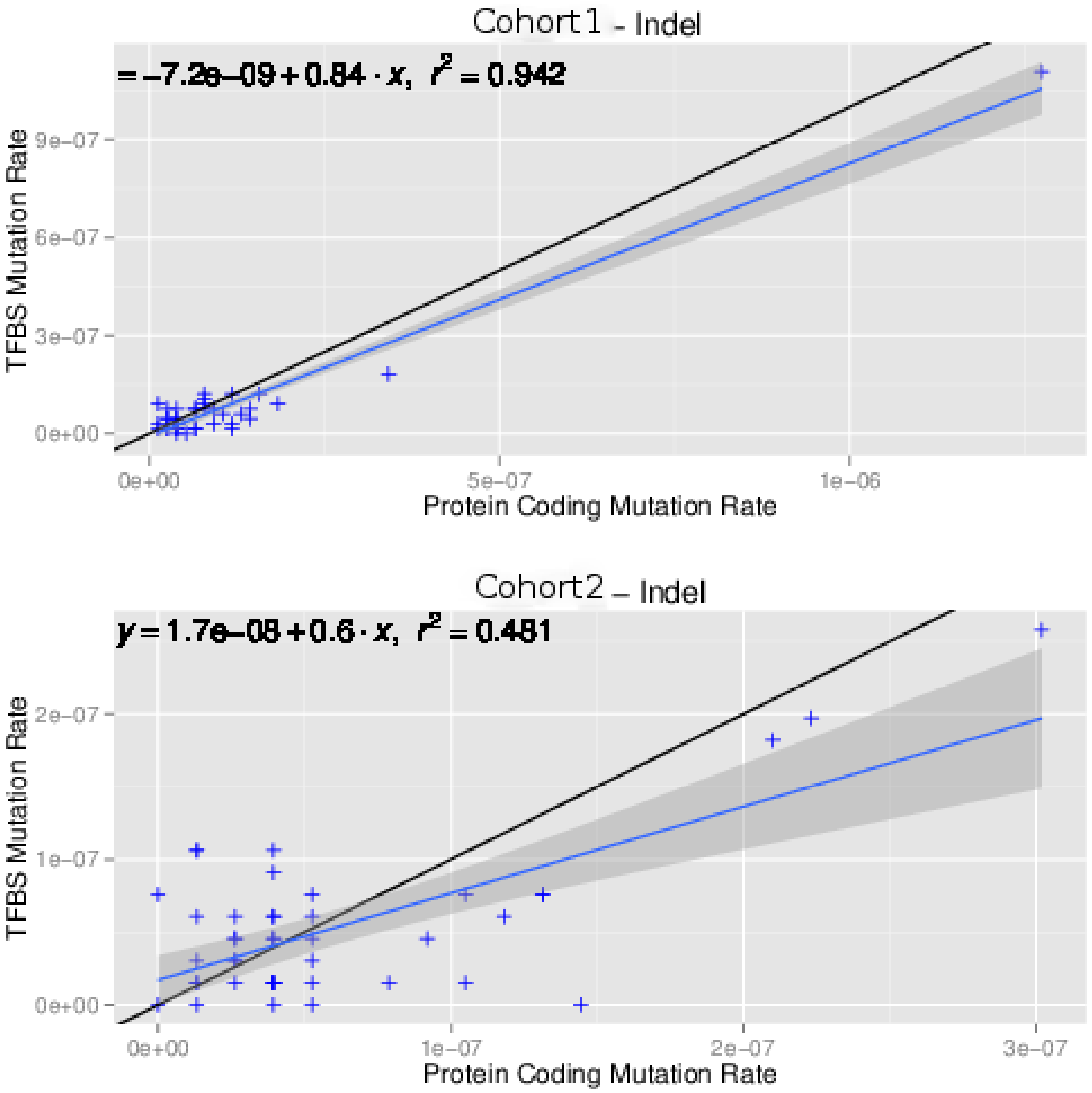

Additional file 1: Figure S2 – Comparison of the indel mutation rates in the *cis*-regulatory and protein-coding spaces. Only indels from cohort1 (**top**) and cohort2 (**bottom**) have been considered. TFBS mutation rates (y-axis) and protein-coding mutation rates (x-axis) were plotted for all the samples in cohort1 (**top**) and cohort2 (**bottom**). Each triangle represents a sample and is color-coded depending on the tumour subtype as in Figure 1 in the main manuscript. Dashed grey lines represent the identity function ($x = y$). Blue lines represent the linear regressions computed from the samples in the two data sets. The equations corresponding to the linear regressions ($y \sim x$) are written on top of the plots along with the computed r^2 statistical measures. Dark grey areas surrounding the blue lines provide the 95% confidence region. Note that the same x- and y-axis scales have been used for both cohort1 (**top**) and cohort2 (**bottom**) plots.

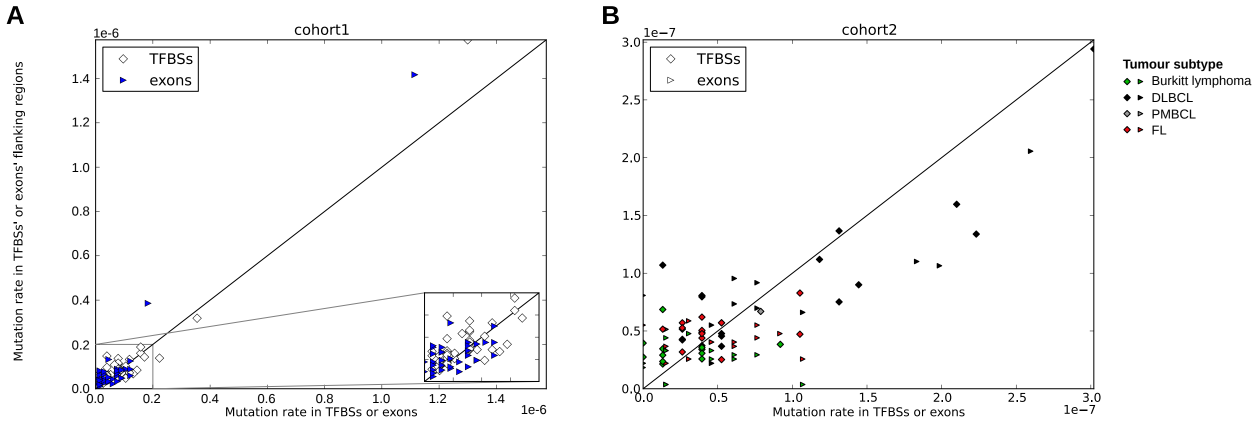

Additional file 1: Figure S3 – Local indel mutation rates for TFBSs and protein-coding exons. Mutation rates are plotted for TFBSs and exons (x-axis) versus their 1kb flanking regions on both sides (y-axis). Each sample from cohort1 (**A**) and cohort2 (**B**) is represented by a square for mutation rates in TFBSs (and their flanking regions) and a triangle for mutation rates in exons (and their flanking regions). Tumor subtypes are color-coded (see legend) like in Figure 1 in the main manuscript. Results are obtained by considering indels. Figures corresponding to SNV local mutation rates are provided in Figure 3 of the main manuscript.

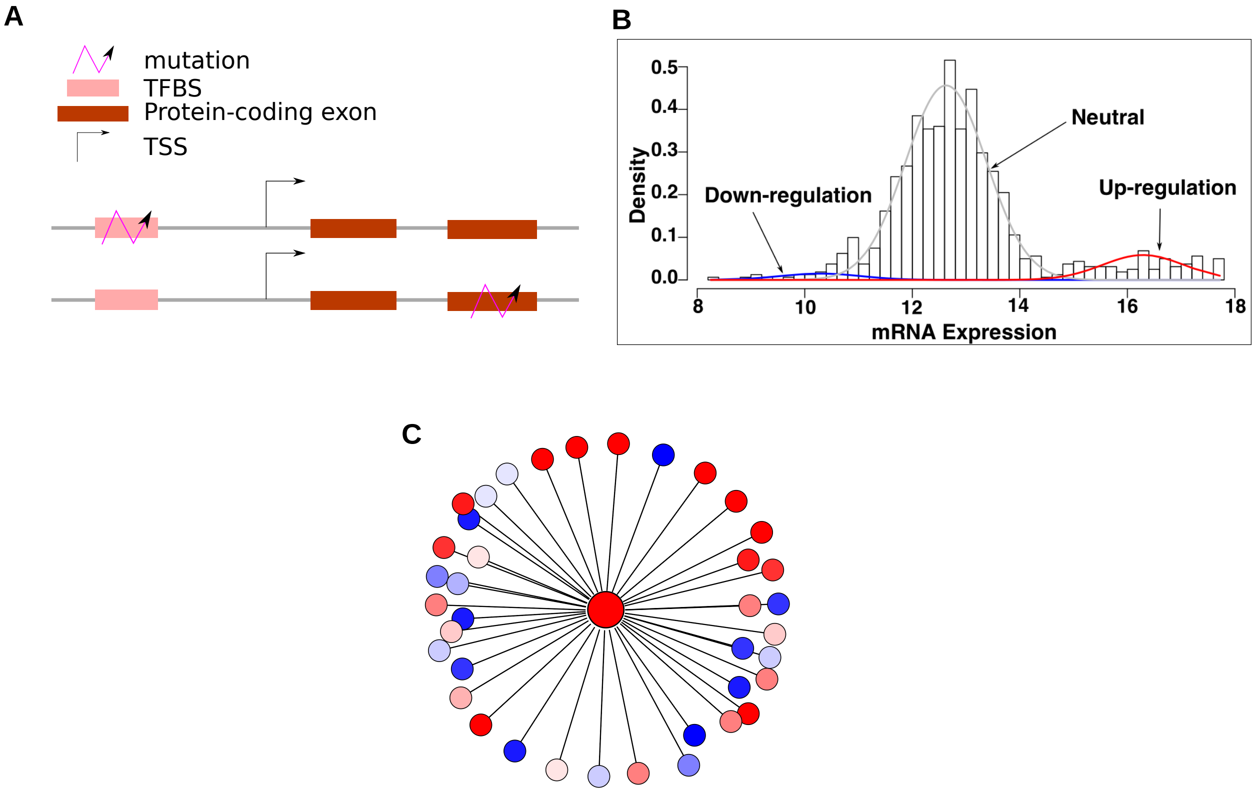

Additional file 1: Figure S4 – xseq input. For running xseq, we considered both mutations lying within protein-coding exons and within TFBSs (**A**). RNA-seq data from all samples in the cohort have been considered to computed down- and up-regulation of genes (**B**). Note that mutations overlapping TFBSs (**A**) are considered only if their closest gene is found down- or up-regulated (**B**) in the considered sample (see Material and Methods). Finally, biological networks (**C**) have been used by xseq to assess the cascading effect of the observed altered expression (nodes in red represent up-regulation and nodes in blue represent down-regulation).

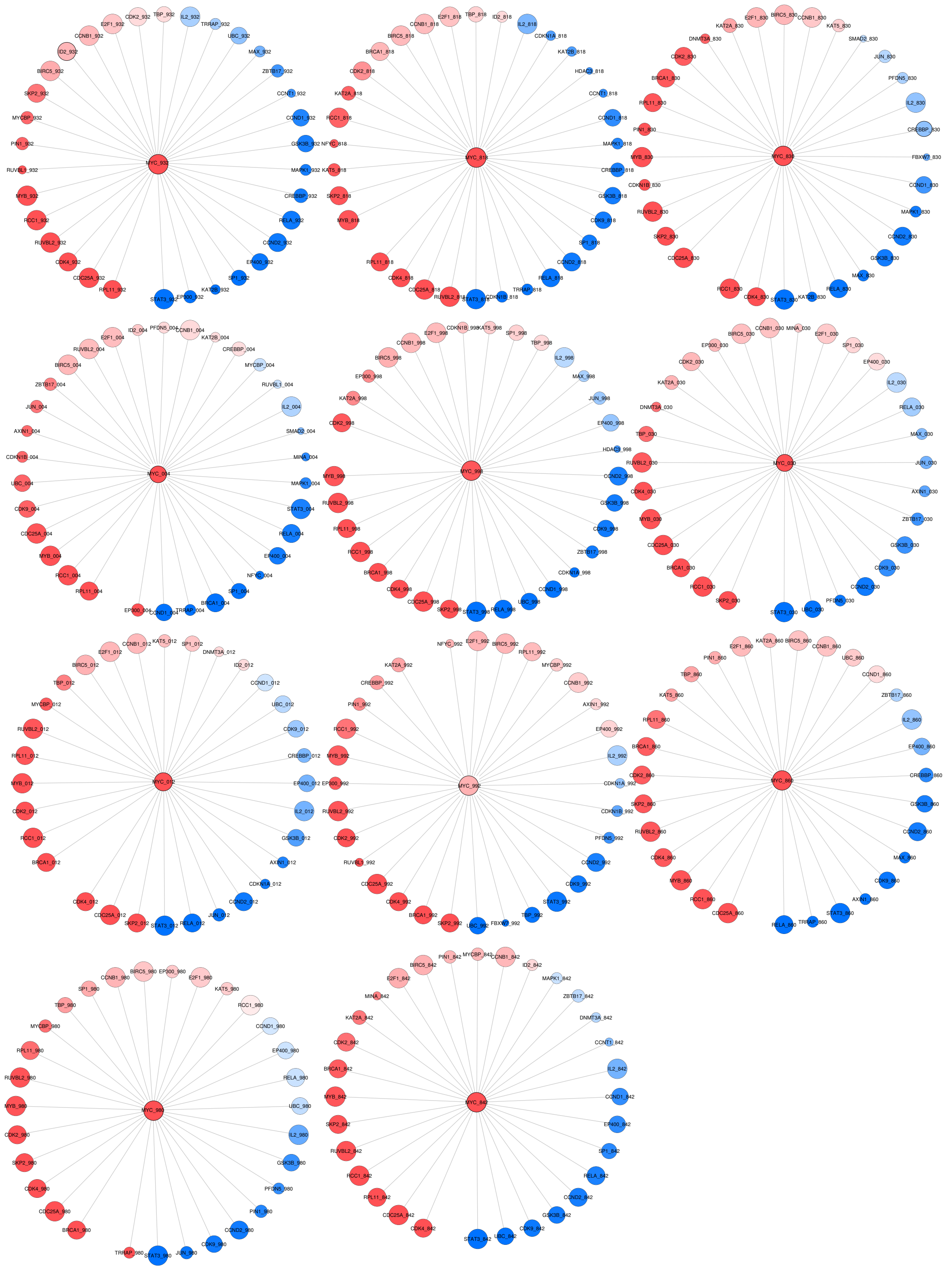

Additional file 1: Figure S5 – MYC expression alteration impact in Burkitt lymphomas. For all the 11 Burkitt lymphoma samples where MYC is predicted as a mutated gene with altered expression by xseq, we provide the gene interactors (from biological networks) predicted as up- (red) or down-regulated (blue). MYC is shown to be up-regulated (red) in all samples. Nodes represent genes and are labeled with the gene name and the sample number (e.g. MYC_932 for the MYC gene in sample SA320**932**).

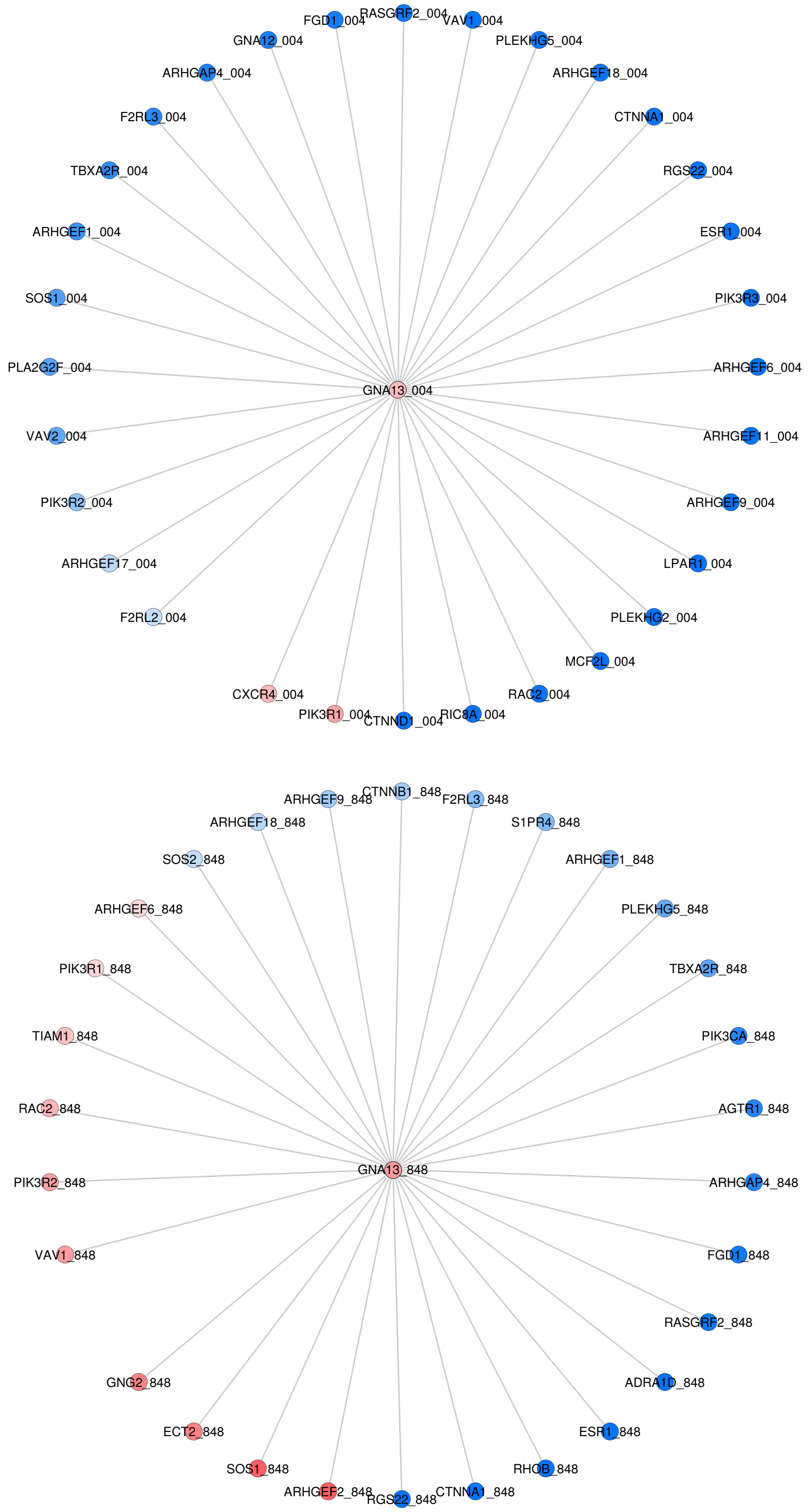

Additional file 1: Figure S6 – GNA13 expression alteration impact. GNA13 is predicted as a mutated gene with altered expression by xseq in samples SA321004 (denoted 004 in the figure) and SA320848 (denoted 848 in the figure). As in Additional Figure 5, nodes represent genes and colors represent either down- (blue) or up-regulation (red).

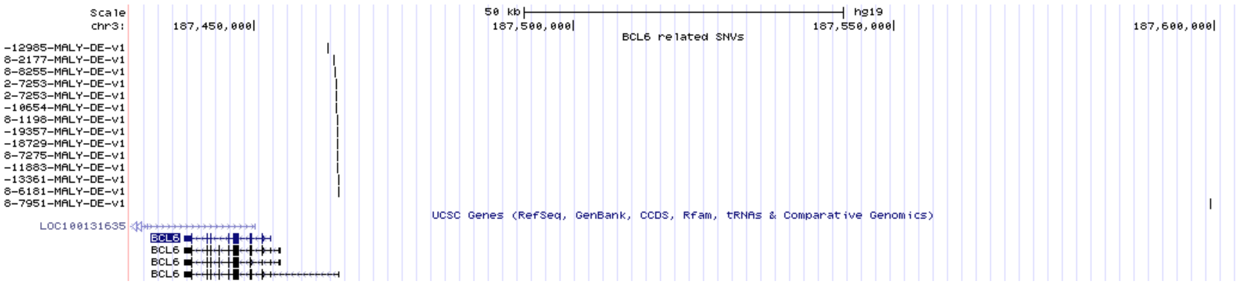

Additional file 1: Figure S7 – Mutations associated to BCL6. Screenshot of the UCSC genome browser highlighting the mutations associated to BCL6 and predicted to alter BCL6 expression by xseq. The upper track provides the localization of the 14 corresponding mutations found in the 4 samples. All the mutations overlap TFBSs. BCL6 gene location is provided in the lower track to highlight that all but one mutations are found close to BCL6 TSS.

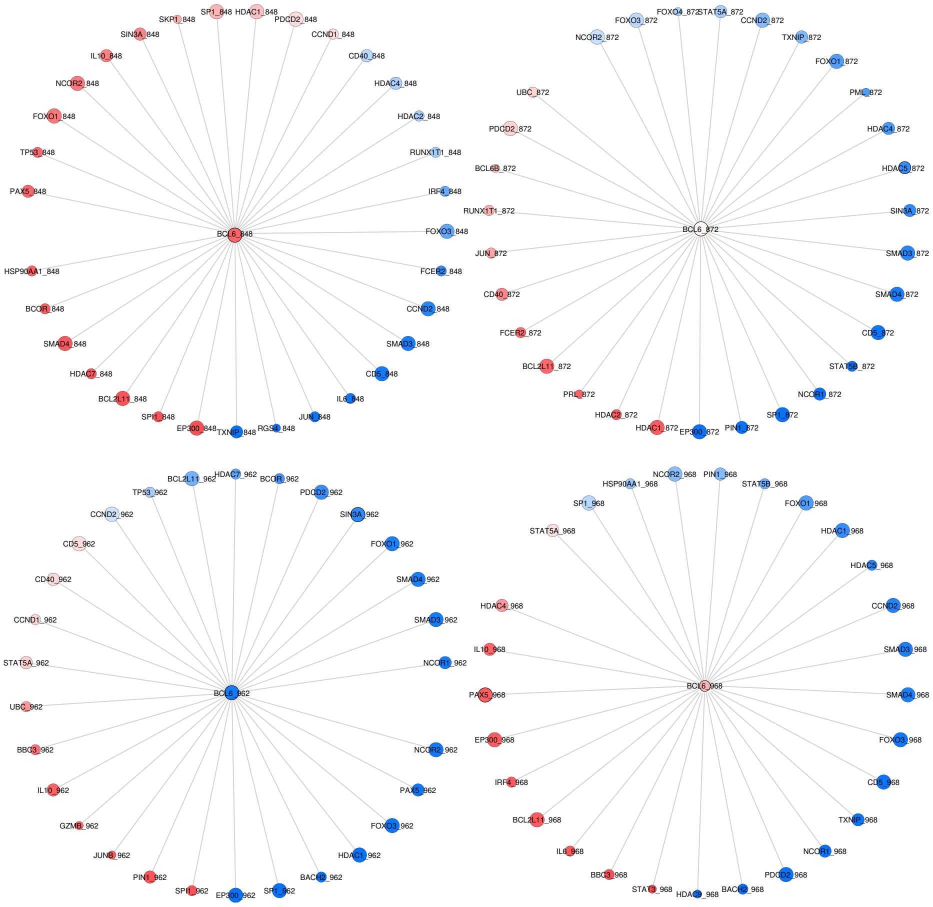

Additional file 1: Figure S8 – BCL6 expression alteration impact. Plots similar to Additional Figures 5-6 describing the gene expression alteration of BCL6 and its interactors in samples SA320848 (denoted 848), SA320872 (denoted 872), SA320962 (denoted 962), and SA320968 (denoted 962).

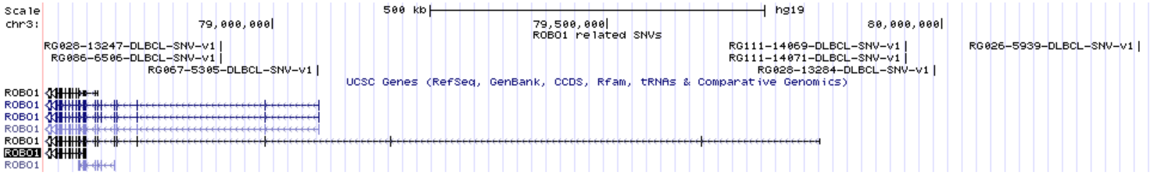

Additional file 1: Figure S9 – Mutations associated to ROBO1. Screenshot of the UCSC genome browser highlighting the mutations associated to ROBO1 and predicted to alter ROBO1 expression by xseq. The upper track provides the localization of the 7 corresponding mutations found in the 4 samples. All the mutations overlap TFBSs. ROBO1 gene location is provided in the lower track.

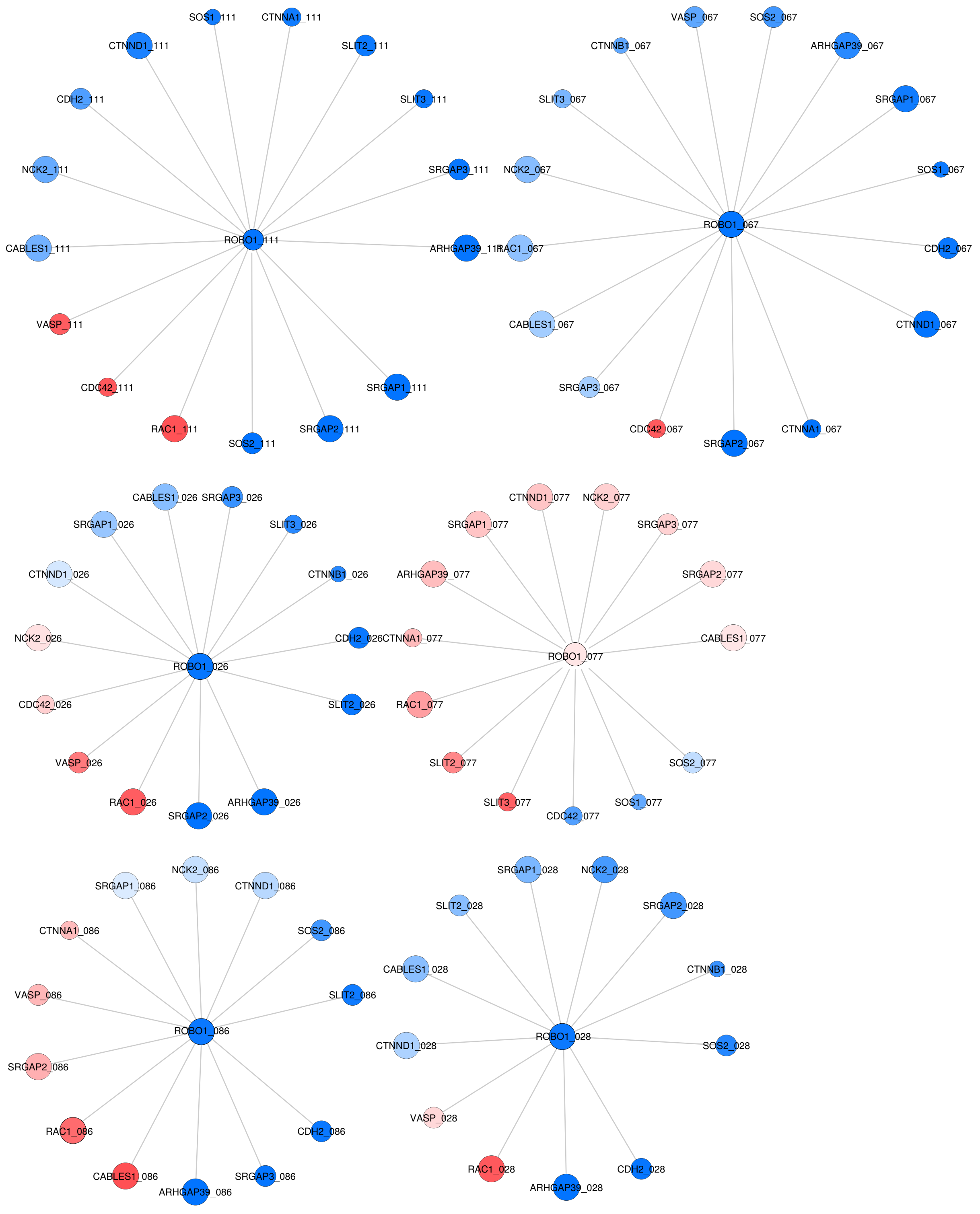

Additional file 1: Figure S10 – ROBO1 expression alteration impact. Plots similar to Additional Figures 5,6,8 describing the gene expression alteration of ROBO1 and its interactors in samples RG111 (denoted 111), RG067 (denoted 067), RG026 (denoted 026), RG077 (denoted 077), RG086 (denoted 086), and RG028 (denoted 028).

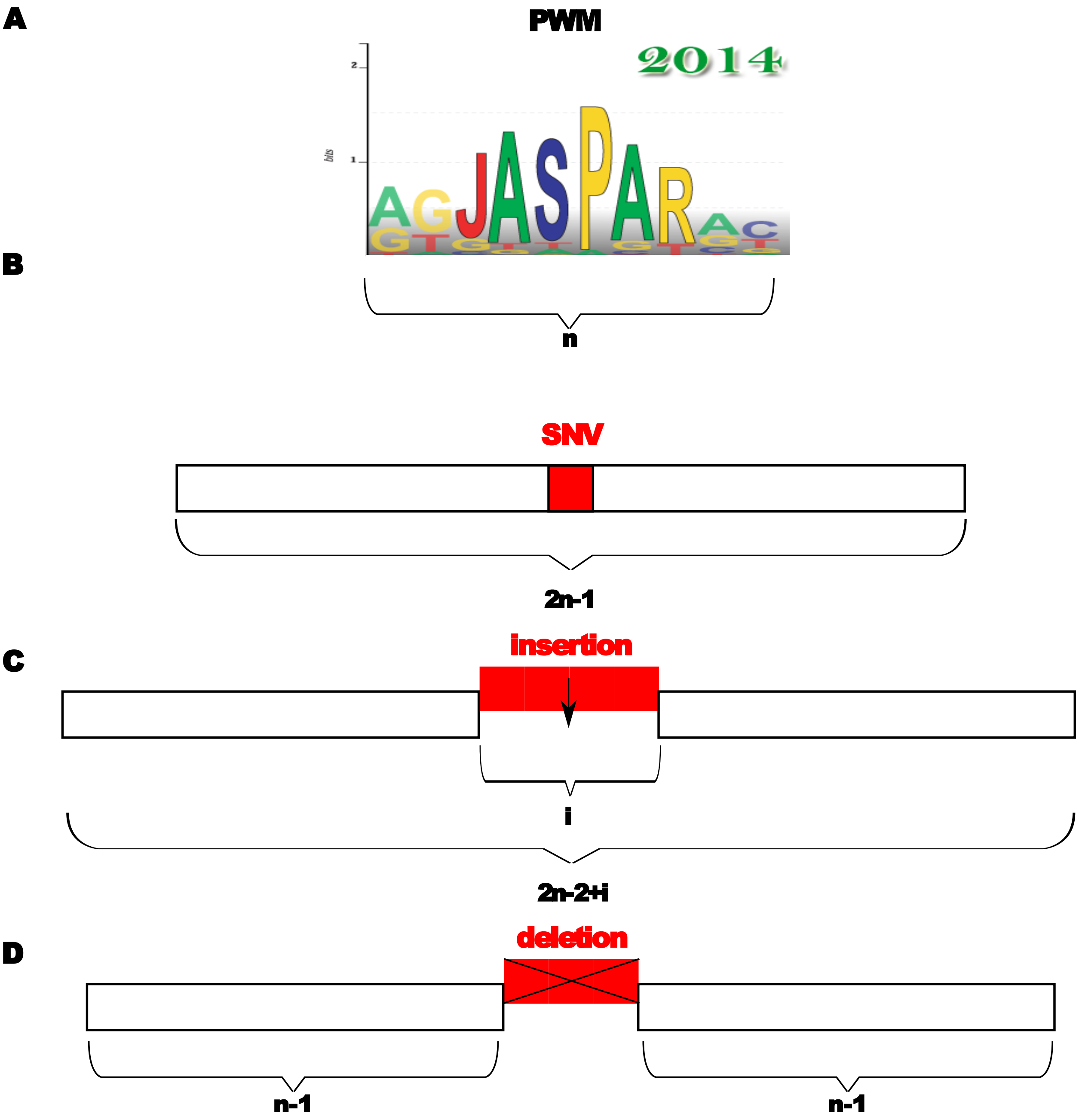

Additional file 1: Figure S11 – Computation of the "best" TFBS after mutation. **A**. JASPAR profiles (derived PWMs) were used to predict TFBSs in altered DNA sequences following a somatic mutation. Let assume the considered PWM of length n. **B**. When a SNV is considered, the PWM scan the sequence of length $2n-1$ centred at the SNV position. **C**. When considering an insertion of i bp, the PWM scans all the sequences of length n overlapping at least one nucleotide of the insertion, leading to a scanned region of $2n-2+i$ nt. **D**. When considering a deletion, the PWM scans the resulting region composed of $n-1$ nt on both sides of the deleted section.

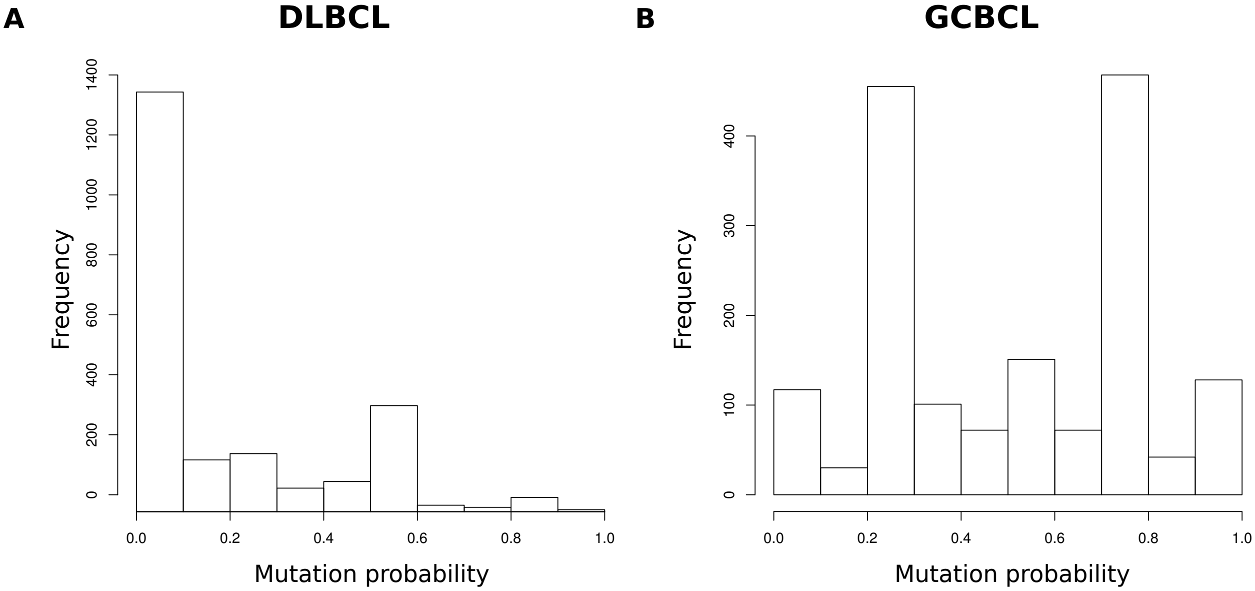

Additional file 1: Figure S12 – Distributions of the xseq probabilities. Distributions of the xseq probabilities for the mutations analyzed in cohort1 (**A**) and cohort2 (**B**).
