# Supplementary material for: Cis-regulatory somatic mutations and gene-expression alteration in B-cell lymphomas
Source: Genome Biol. 2015 Apr 23;16(1):84. doi: 10.1186/s13059-015-0648-7 (PMC4467049; doi:10.1186/s13059-015-0648-7)
Supplement: Additional file 3 — Case examples. Details of the case examples predicted by xseq (HAS2, GNA13, BCL6 and ROBO1). [file 13059_2015_648_MOESM3_ESM.pdf]

HAS2

HAS2 is an essential membrane-embedded Hyaluronan (HA) synthase necessary for the catalyzation of HA [1]. We predicted HAS2 to be associated with mutations and altered expression in 4 samples from cohort1. Two samples (RG014 and RG027) were associated with mutations in the protein coding space whereas two others (RG067 and RG116) were associated to mutations in TFBSs. The RG116 sample harboured a SNV in a CEBPA TFBS that was predicted to be deleterious (see Figure 8ABCDE from the manuscript). We observed a down-regulation of the HAS2 gene expression in the sample associated to the disruption of the CEBPA TFBS. From the four samples where HAS2 was predicted by xseq, we observed that two of them showed a down-regulation and the two others an up-regulation. Note that HAS2 has been described previously as either an oncogene or a tumour suppressor depending on HA length and concentration in the extracellular matrix [1].

GNA13

The G-protein subunit $\alpha - 13$ (GNA13) has been previously linked to tumour progression and has been described as an important mediator of prostate cancer cell invasion [2]. It has also been previously identified as a recurrent target of mutation with inactivation impact in DLBCLs [3]. In our xseq analysis of cohort2, GNA13 was predicted to be mutated with altered expression in two Burkitt lymphoma samples. One sample (SA321004) was associated to a SNV in the protein-coding space whereas another one (SA32848) was associated to a SNV lying within three overlapping TFBSs (for the TFs GATA1, GATA3, and JUND). We predicted the SNV as disrupting the TFBS associated to GATA3. Given the GATA3 TF binding profile, we observed that the mutation was one of the most severe mutation that can occur to the TFBS (see Figure 8FGHIJ from the manuscript). In the corresponding sample, we observed an up-regulation of GNA13 transcription consistent with an important role of GNA13 for cancer cell invasion. Notice that GNA13 was also found up-regulated when associated to a mutation in the sample SA321004 associated to a mutation in an exon (Additional file 1: Figure S6).

The Burkitt lymphoma SA320848 sample highlighted a deregulation of expression of genes known to interact with GNA13 (see Additional file 1: Figure S6). We observed the up-regulation of the known oncogenes VAV1 [4] and ECT2 [5]. Moreover, the RHOB gene was down-regulated and has been shown to associate with cell proliferation and an increase in DNA double strand breaks [6].

BCL6

BCL6 was predicted with altered expression associated to mutations in four samples (one corresponding to a Burkitt lymphoma and three to DLBCLs) from cohort2. Three of the altered expression were associated to mutations in TFBSs (two of which were predicted to be disruptive) and one to a missense mutation in a protein-coding exon. Interestingly, we observed an up-regulation of BCL6 in samples SA320848 and SA320968 which were associated to mutations disrupting TFBSs. On the contrary, a down-regulation was observed in SA320962 associated to mutations in TFBSs (not predicted to be disruptive) and no expression change was observed in SA320872 associated to a mutation in an exon. All the mutations but one overlapping TFBSs and associated to BCL6 in the corresponding samples were found in the promoter region of BCL6 (see Additional file 1: Figure S7). In sample SA320962, a mutation was found to lie within two predicted overlapping STAT3 TFBSs. The mutation did not seem to disrupt the TFBSs.

The up-regulation of BCL6 in the two samples was associated to several mutations in TFBSs (7 SNVs in SA320848 lied within 10 TFBSs, 3 of which predicted to be deleterious; 5 SNVs in SA320968 lied within 8 TFBSs, 1 of which predicted to be deleterious). Three SNVs in SA320848 were predicted to disrupt TFBSs of the TFs BRCA1, E2F1, USF1, and BHLHE40 (a mutation overlaps two TFBSs). In SA320968, a SNV was predicted to disrupt a GATA3 TFBS. The presence of multiple SNVs lying within TFBSs in the core promoter of the BCL6 gene is an indication of a potential deregulation of BCL6 expression through the

alteration of transcriptional regulation. The absence of mutation within the exonic regions in the same samples is supporting this hypothesis.

We next focused on the two disruptive TFBS samples to determine the potential cascading effect arising from the up-regulation of BCL6 in SA320848 and SA320968. Over expression of BCL6, an oncogene, enables for accelerated proliferation and makes the cell more lenient to DNA damage [7]. Both SA320848 and SA320968 samples showed a down-regulation of SMAD3 or SMAD4 and TXNIP which are all known to have biological interactions with BCL6 (see Additional file 1: Figure S8). The DLBCL sample SA320968 presented a down-regulation of SMAD3 and SMAD4 whereas the Burkitt lymphoma sample SA320848 displayed a down-regulation of SMAD3 (see Additional file 1: Figure S8). Over expression of BCL6 also inhibited the tumour suppressors SMAD3 and SMAD4. TXNIP was down-regulated in both samples. The down-regulation of TXNIP has already been observed in many cancer types including DLBCLs, and has shown to be a tumour suppressor in thyroid cancer [7].

ROBO1

Our approach allows for extracting genes for which little is known regarding their involvement in cancer. For instance, ROBO1, a transmembrane protein, was predicted with altered expression associated with mutations in six samples from cohort1 (see Figure 6 from the manuscript and Additional file 1: Figure S9). Five of these samples highlighted mutations in TFBSs and one sample was associated to a mutation in the protein-coding space. ROBO1 has been previously characterized as a potential tumour suppressor in different types of cancer [8, 9, 10]. Another study highlighted a tumour specific hypermethylation of the promoter region of the gene in breast tumours, repressing the transcription of the ROBO1 gene [9]. Here, we found that ROBO1 was down-regulated in the five samples associated to mutations in TFBSs whereas it was slightly up-regulated in the sample harbouring a mutation in the protein-coding region. Even though we did not predict any of the mutations to disrupt the overlapped TFBSs, they might impact the binding of the TFs or even create new TFBSs for competing TFs.

To identify the potential impact of the mutated predicted tumour suppressor ROBO1, we inspected the expression deregulation of known interacting genes. We focused on genes repeatedly shown to be deregulated when ROBO1 was predicted to be deregulated by mutations. Four out of the five DLBCL samples where ROBO1 was down-regulated and harbouring TFBS mutations exhibited either SOS1 or SOS2 as down-regulated (see Additional file 1: Figure S10), which are Son of Sevenless (SOS) genes known to be involved in the Raf/Ras/MEK/ERK/MAPK pathway where the interaction of Ras with GDP/GTP-exchange factors is mediated by SOS [11]. Another ROBO1 interacting gene commonly deregulated in association with ROBO1 was RAC1. RAC1 has a high probability of being up-regulated in four out of the five samples where ROBO1 was associated to TFBS mutations; one other sample harboured a missense protein coding mutation in RAC1 (see Additional file 1: Figure S10). [8] showed that RAC1 was activated when ROBO1 was lost, inducing cell migration in lymphoma cells. This observation is in agreement with the up-regulation of RAC1 combined with the down-regulation of ROBO1 in all but one sample where ROBO1 was predicted to be associated to mutations within TFBSs. All these results taken together shed light into the supposed tumour suppressor role of the ROBO1 gene. We highlighted here that ROBO1 might be down-regulated at the transcriptional level by mutations in *cis*-regulatory elements while no mutations were found in the protein-coding space.

References

[1] Escudero, D.: HAS2 (hyaluronan synthase 2). Atlas of Genetics and Cytogenetics in Oncology and Haematology **14**(2), 121–123 (2010). doi:10.4267/2042/44682. Accessed 2014-07-12

[2] Rasheed, S.A.K., Teo, C.R., Beillard, E.J., Voorhoeve, P.M., Casey, P.J.: MicroRNA-182 and microRNA-200a control g-protein subunit α-13 (GNA13) expression and cell invasion synergistically in prostate cancer cells. The Journal of Biological Chemistry **288**(11), 7986–7995 (2013). doi:10.1074/jbc.M112.437749

[3] Morin, R.D., Mendez-Lago, M., Mungall, A.J., Goya, R., Mungall, K.L., Corbett, R.D., Johnson, N.A., Severson, T.M., Chiu, R., Field, M., Jackman, S., Krzywinski, M., Scott, D.W., Trinh, D.L., Tamura-Wells, J., Li, S., Firme, M.R., Rogic, S., Griffith, M., Chan, S., Yakovenko, O., Meyer, I.M., Zhao, E.Y., Smailus, D., Moksa, M., Chittaranjan, S., Rimsza, L., Brooks-Wilson, A., Spinelli, J.J., Ben-Neriah, S., Meissner, B., Woolcock, B., Boyle, M., McDonald, H., Tam, A., Zhao, Y., Delaney, A., Zeng, T., Tse, K., Butterfield, Y., Birol, I., Holt, R., Schein, J., Horsman, D.E., Moore, R., Jones, S.J.M., Connors, J.M., Hirst, M., Gascoyne, R.D., Marra, M.A.: Frequent mutation of histone-modifying genes in non-hodgkin lymphoma. Nature **476**(7360), 298–303 (2011). doi:10.1038/nature10351

[4] Wakahashi, S., Sudo, T., Oka, N., Ueno, S., Yamaguchi, S., Fujiwara, K., Ohbayashi, C., Nishimura, R.: VAV1 represses e-cadherin expression through the transactivation of snail and slug: a potential mechanism for aberrant epithelial to mesenchymal transition in human epithelial ovarian cancer. Translational Research: The Journal of Laboratory and Clinical Medicine **162**(3), 181–190 (2013). doi:10.1016/j.trsl.2013.06.005

[5] Greer, E.R., Chao, A.T., Bejsovec, A.: Pebble/ECT2 RhoGEF negatively regulates the wingless/wnt signaling pathway. Development (Cambridge, England) **140**(24), 4937–4946 (2013). doi:10.1242/dev.101303

[6] Meyer, N., Peyret-Lacombe, A., Canguilhem, B., MÃl'dale-Giamarchi, C., Mamouni, K., Cristini, A., Monferran, S., Lamant, L., Filleron, T., Pradines, A., Sordet, O., Favre, G.: RhoB promotes cancer initiation by protecting keratinocytes from UVB-induced apoptosis but limits tumor aggressiveness. The Journal of Investigative Dermatology **134**(1), 203–212 (2014). doi:10.1038/jid.2013.278

[7] Huang, C., Hatzi, K., Melnick, A.: Lineage-specific functions of bcl-6 in immunity and inflammation are mediated by distinct biochemical mechanisms. Nature Immunology **14**(4), 380–388 (2013). doi:10.1038/ni.2543

[8] Parray, A., Siddique, H.R., Kuriger, J.K., Mishra, S.K., Rhim, J.S., Nelson, H.H., Aburatani, H., Konety, B.R., Koochekpour, S., Saleem, M.: ROBO1, a tumor suppressor and critical molecular barrier for localized tumor cells to acquire invasive phenotype: Study in african-american and caucasian prostate cancer models. International Journal of Cancer. Journal International Du Cancer (2014). doi:10.1002/ijc.28919

[9] Xian, J., Aitchison, A., Bobrow, L., Corbett, G., Pannell, R., Rabbitts, T., Rabbitts, P.: Targeted disruption of the 3p12 gene, dutt1/robo1, predisposes mice to lung adenocarcinomas and lymphomas with methylation of the gene promoter. Cancer Research **64**(18), 6432–6437 (2004). doi:10.1158/0008-5472.CAN-04-2561

[10] Bonner, A.E., Lemon, W.J., You, M.: Gene expression signatures identify novel regulatory pathways during murine lung development: implications for lung tumorigenesis. Journal of Medical Genetics **40**(6), 408–417 (2003)

[11] Orton, R.J., Sturm, O.E., Gormand, A., Wolch, W., Gilbert, D.R.: Computational modelling reveals feedback redundancy within the epidermal growth factor receptor/extracellular-signal regulated kinase signalling pathway. IET systems biology **2**(4), 173–183 (2008). doi:10.1049/iet-syb:20070066
